# EnDeep4mC predicts DNA *N*^4^-methylcytosine sites using a dual-adaptive feature encoding framework in deep ensembles

**DOI:** 10.1101/gr.280977.125

**Published:** 2026-03

**Authors:** Shuyu Zhang, Quan Zou, Mengting Niu, Zhibin Lv, Antony Stalin, Ximei Luo

**Affiliations:** 1 Institute of Fundamental and Frontier Sciences, University of Electronic Science and Technology of China, Chengdu 610054, Sichuan, China;; 2 Yangtze Delta Region Institute (Quzhou), University of Electronic Science and Technology of China, Quzhou 324000, Zhejiang, China;; 3 College of Biomedical Engineering, Sichuan University, Chengdu 610041, Sichuan, China

## Abstract

DNA *N*^4^-methylcytosine (4mC), a key epigenetic modification regulating DNA repair and replication, requires efficient computational detection methods due to experimental limitations. Although machine learning predictors have been proposed, their performance could be enhanced through systematic optimization of feature encoding schemes. Here, we propose EnDeep4mC, a dual-adaptive framework integrating species-specific modeling with ensemble deep learning architectures to systematically optimize feature encoding schemes. Evaluated across six species, EnDeep4mC demonstrates commendable prediction performance and significantly outperforms current state-of-the-art predictors. Cross-species validation confirms its robust transferability from animal to microbe groups. Evolutionary analysis further uncovers the functional differentiation of 4mC sequences in biological evolution: Prokaryotic 4mC relies on stable patterns, whereas eukaryotes achieve regulatory plasticity through dynamic sequence combinations, which provides experimental evidence for species-adaptive encoding strategies.

DNA methylation is a form of DNA epigenetic modification, which modulates the genetic performance without changing the DNA sequence (Moore et al. 2013). Numerous studies have shown that DNA methylation can alter chromatin structure, DNA conformation, DNA stability, and DNA–protein interaction to regulate gene expression (Razin and Riggs 1980; Cheng 1995; Jones and Takai 2001; Robertson 2005; Wang et al. 2024b). *N*^6^-methyladenine (6mA), 5-methylcytosine (5mC), and *N*^4^-methylcytosine (4mC) are three common DNA methylation modifications. Compared with 5mC and 6mA, 4mC has received comparatively less attention, primarily owing to existing costly and time-consuming experimental methods (Flusberg et al. 2010; Yu et al. 2015). First identified in 1983, 4mC is ubiquitous in prokaryotes (such as bacteria) but relatively rare in eukaryotes(Beaulaurier et al. 2019). Similar to 6mA and 5mC, 4mC plays critical roles in many biological processes, including DNA repair and replication. It operates as a core component in the antiphage immune system of bacteria, mainly reflected in the participation of 4mC in the bacterial restriction–modification system (RM system). This system safeguards bacteria against phage and other invasive foreign DNA (O'Brown et al. 2019; Yu et al. 2021; Xiong et al. 2022). Consequently, 4mC is recognized as a key regulator of gene expression, DNA repair, and antiphage immunity in prokaryotes, particularly bacteria, highlighting its potential value for microbiology and antimicrobial drug development (Vandenbussche et al. 2021; Yu et al. 2023; Zheng et al. 2023). To systematically investigate its biological functions and mechanisms, developing reliable detection methods is essential. Although recent advances in nanopore sequencing allow for the direct detection of 5mC and 4mC during basecalling (Liu et al. 2021b; Wang et al. 2021), conventional experimental approaches remain expensive, time consuming, and labor intensive (Flusberg et al. 2010; Yu et al. 2015). It is also important to note that nanopore-based detection methods depend on machine learning (ML) models to interpret sequencing signals accurately (Liu et al. 2019; Wang et al. 2021; Chen et al. 2025; Galeone et al. 2025). Therefore, developing bioinformatics tools for the large-scale, accurate, and efficient 4mC site identification is a growing trend and an effective supplement to laboratory methods (Wang et al. 2024a).

In recent years, considerable progress has been made in predicting DNA methylation sites. Researchers have developed a series of effective DNA methylation site predictors based on traditional ML and recent deep learning (DL) algorithms (Feng et al. 2016; Zhou et al. 2016; Jin et al. 2019; Bonet et al. 2022). Among them, Chen et al. (2017) proposed the first SVM-based predictor iDNA4mC and screened the MethSMRT database (Ye et al. 2017) to construct the benchmark data set. Most of the subsequent studies based on ML algorithms also continued to adopt this data set, such as 4mCPred (He et al. 2019), Meta-4mCpred (Manavalan et al. 2019), 4mcPred-SVM (Wei et al. 2019a), 4mcPred-IFL (Wei et al. 2019b), etc. Subsequently, Khanal et al. (2019) proposed 4mCCNN, the first predictor based on the DL method, convolutional neural networks (CNN), for 4mC identification. Based on this, Liu et al. (2021a) proposed DeepTorrent by combining multiple feature encoding schemes with CNN and Bi-LSTM algorithms. Xu et al. (2021) proposed Deep4mC to further improve the model performance by building a stacked model based on the prediction probabilities of six different ML algorithms. Liu et al. (2022) addressed a sample imbalance of species via scale-aware learning, integrating class weights into cross-entropy loss to develop MSNet-4mC. Based on Hyb4mC (Liang et al. 2022), iDNA-MS (Lv et al. 2020), and DeepTorrent (Liu et al. 2021a), Li et al. (2023) constructed a more robust data set and proposed EpiTEAmDNA, the first stacked model integrating traditional ML algorithms and CNN. It achieved better performance than other DL-based algorithms on small sample data sets. Yao et al. (2024) used a new data set proposed by Zeng et al. (Zeng and Liao 2020) to leverage existing models through transfer learning and combined multiple ensemble learning techniques to build DeepSF-4mC, which achieved enhanced prediction performance on three specific species.

However, the above methods still exhibit two critical constraints. First, traditional methods usually adopt static encoding schemes in feature engineering that neglect the distinct characteristics of different species and models, resulting in the one-size-fits-all encoding dilemma. Second, prevalent predictors mostly rely on homogeneous architectures, which are difficult in simultaneously capturing local conserved patterns, long-range context dependence, and global attention correlations of DNA sequences.

To address these limitations, we propose EnDeep4mC, a dual-adaptive optimization framework. The core innovations of EnDeep4mC include (1) a species-model collaborative mechanism that dynamically selecting feature encodings via cross-species/model performance quantification and incremental feature selection and (2) a three-tier probability fusion architecture in which Tier-I employs models (CNN, Bi-LSTM, Transformer) to parallelly generate prediction initial probabilities, Tier-II concatenates probabilities via XGBoost/LightGBM for feature enhancement, and Tier-III applies elastic-net logistic regression to achieve a robust and regularized decision fusion.

Compared with existing predictors, EnDeep4mC achieves three advances: (1) It transforms traditional feature selection into a dual-optimization problem integrating species specificity and model architecture, thereby overcoming the fixed-feature limitations in cross-species/model scenarios; (2) it achieves 94.72% average AUC across benchmark data sets, outperforming SOTA predictors with robust cross-species transferability; and (3) it systematically elucidates species-feature associations and reveals 4mC sequence functional differentiation in biological evolution from a eukaryote/prokaryote perspective, offering potential biological insights that could guide future adaptive encoding strategies.

The following sections first detail the Methods used to construct the EnDeep4mC framework and then present a comprehensive evaluation of its performance and biological findings.

## Methods

### Benchmark data sets

A variety of benchmark data sets have been proposed in the field of 4mC site prediction. Most of them before 2021 were designed for ML models with relatively few samples (Chen et al. 2017; Ye et al. 2017; He et al. 2019; Manavalan et al. 2019; Wei et al. 2019a,b). However, reasonable and sufficient data sets are essential for training DL models. The 4mC data used in this experiment are from EpiTEAmDNA (Li et al. 2023). To the best of our knowledge, this is the most comprehensive resource of its kind. This data set contains three modification types: 4mC, 5mC, and 6mA. The 4mC modification data are integrated from data sets established in Hyb4mC (Liang et al. 2022), iDNA-MS (Lv et al. 2020), and DeepTorrent (Liu et al. 2021a). All collected 4mC sequences (41 bp) have a methylated cytosine (C) at the center, whereas negative samples are nonmethylated, as confirmed by SMRT sequencing. All input DNA sequences are processed into 41 bp fixed-length samples. Sequences <41 bp are symmetrically padded with N's. Longer sequences are segmented into consecutive, overlapping 41 bp sliding windows. This ensures the model operates within its validated parameters (Supplemental Fig. S1; for details, see Supplemental Methods). Furthermore, to ensure the consistency of comparison, the CD-HIT tool was used to remove redundant sequences. The final data set comprises 11 model organisms. For model training, we mainly focused on six species: *Arabidopsis thaliana*, *Caenorhabditis elegans*, *Drosophila melanogaster*, *Escherichia coli*, *Geobacillus subterraneus*, *Geobacter pickeringii*. The respective positive sample counts for these species are 125,628, 68,917, 109,289, 2068, 14,877, and 5687, respectively. Training and independent test data sets were split as shown in Supplemental Table S1.

### Overview of the EnDeep4mC architecture

**Figure 1.**
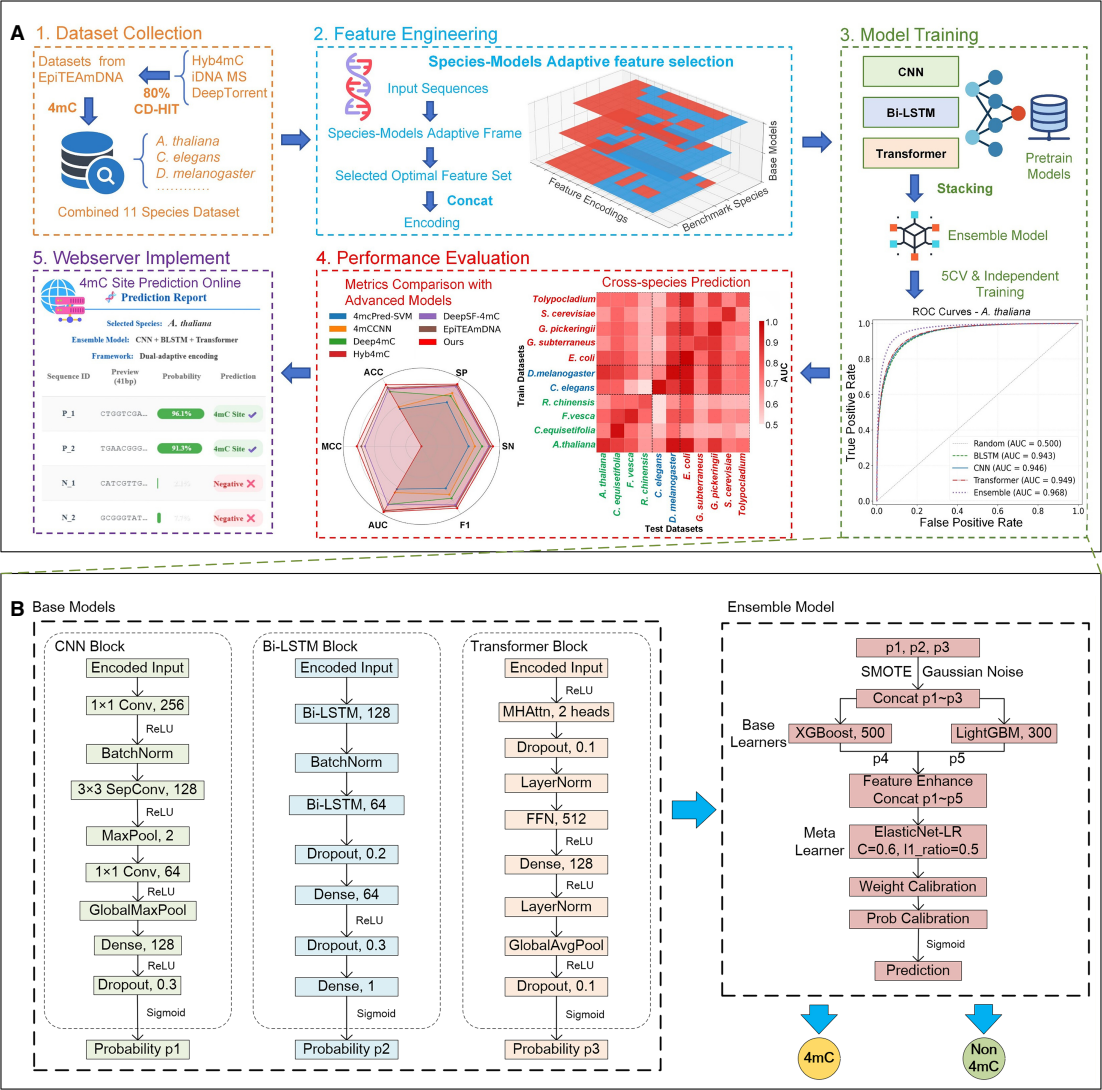
The overall framework of EnDeep4mC. (*A*) Main workflow of the experiment. (*B*) Model architecture diagrams of the base model CNN, Bi-LSTM, Transformer, and ensemble model.

The EnDeep4mC framework employs a dual-phase architecture for species-model co-optimization: (1) a dynamic feature selection (DFS) module identifies optimal species-model feature combinations via dual adaptation, and (2) a heterogeneous ensemble fusion module constructs a metalearning system using deep probabilistic predictions (Fig. 1B).

The workflow initiates with feature-enhanced base models (CNN, Bi-LSTM, Transformer), each trained on species/model-specific optimized encodings to generate probabilistic outputs. These outputs are hierarchically fused via stacking, processed by XGBoost/LightGBM as base learners and finalized through elastic network logistic regression, to yield robust classification.

### Feature-enhanced base model architecture

Guided by ensemble learning principles that emphasize the importance of error diversity among base models (Dietterich 2000; Kuncheva and Whitaker 2003), we constructed three DL networks—CNN, Bi-LSTM, and Transformer—to collaboratively capture multiscale sequence features (for visual effect, Supplemental Fig. S2). This principled selection, validated by subsequent quantitative diversity analysis (Supplemental Table S2), ensures that the ensemble leverages distinct and complementary predictive behaviors rather than redundant signals.

The CNN model adopts a hierarchical convolution architecture with 256 1D convolution kernels (kernel_size = 1) and separable convolution layers (128 filters, kernel_size = 3). The sequence feature compression is realized by the global max pooling layer. L2 regularization (λ = 0.001) and dropout (rate = 0.3) were introduced to mitigate overfitting.

Bi-LSTM model employs bidirectional recursive structure with two LSTM layers: The first (128 units) captures local temporal patterns via sequential information retention, and the second (64 units) performs feature abstraction. The recurrent dropout (rate = 0.1) and batch normalization were introduced to enhance the generalization.

The Transformer model features a multilayer encoder with multihead attention (two heads) and a feed-forward network (FFN_dim = 64) per layer. Training stability is ensured via layer normalization (LayerNorm $\varepsilon =1\times 10^{-6}$) and adaptive learning rate scheduling (initial lr = 1 × 10^−3^, decay rate = 0.9).

All base models were trained on species-specific data sets using fivefold cross-validation, optimized with Adam (gradient clipping threshold = 1.0) and sigmoid activation. The DFS module was integrated into each base model to dynamically optimize encoding combinations during training.

### Probabilistic guided ensemble model

EnDeep4mC employs a hierarchical probabilistic fusion architecture (Fig. 1B), achieving collaborative optimization through three stages:

1. Multimodal probability generation: CNN, Bi-LSTM, and Transformer—each model optimizes feature encoding through the DFS framework—independently generate prediction probabilities of the samples as their outputs.
2. Base learners layer: The stacking classifier utilizes XGBoost (n_estimators = 500, max_depth = 7) and LightGBM (n_estimators = 300, reg_alpha = 0.2) as base learners to process the 3D probability vectors concatenated from three DL models. Their prediction outputs combine with original probabilities to form the 5D meta feature (three base probabilities + two learner outputs).
3. Meta learner layer: The final decision fusion is realized by elastic network logistic regression. In the regularization strategy, the elastic network constraint (L1 ratio = 0.5, C = 0.6) enables feature selection and overfitting suppression.

To maximize transparency and reproducibility, the complete architectures and hyperparameters for the three base models (CNN, Bi-LSTM, Transformer), the ensemble learners (XGBoost, LightGBM), and the metalearner are documented in Supplemental Tables S3 through S5, respectively. These tables specify all layers, regularization strategies, optimizer settings, and the complete training protocol, enabling exact replication of the model architectures and experimental results.

### Feature encoding schemes

More than 24 common feature encoding schemes are widely used in epigenetic site prediction. In the work of EpiTEAmDNA, Li et al. (2023) demonstrated that 10 feature encoding schemes such as pseKNC, pseDNC, and DAC are unsuitable for the 4mC site prediction task based on DL architecture based on computational efficiency and contribution to accuracy improvement. Ultimately, this study utilizes the remaining 14 feature encoding schemes—ENAC, Binary, NCP, EIIP, *k*-mer, CKSNAP, PseEIIP, TNC, RCK-mer, SCPseTNC, PCPseTNC, ANF, NAC, and TAC—as our candidate set. The key characteristics of the 14 selected encoding schemes are summarized in Supplemental Table S6. Based on the encoding schemes defined in the iLearn toolkit (Chen et al. 2020), we provide their detailed descriptions in the Supplemental Methods.

### DFS framework

To enhance the universality of feature encoding schemes, we proposed a DFS framework to optimize the feature selectivity of species and the characteristics of model architecture simultaneously. As shown in Figure 1A, the framework achieves end-to-end optimization through two stages of feature-model cross-evaluation and dynamic feature fusion. The core innovation of the framework is to establish a 3D selection space of species-model-features and to realize the dynamic adaptation of feature combinations through a two-layer optimization mechanism.

The first stage is feature-model cross-evaluation. The purpose of this stage is to realize the joint analysis of species model traits by constructing a 3D evaluation matrix. Specifically, for each species *s* ∈ {*A*. *thaliana*, *C*. *elegans*, …} and DL base model *k* ∈ {CNN, BLSTM, Transformer}, the utility of 14 candidate feature encoding methods *f*_*i*_ is evaluated independently. The evaluation process was carried out in the following steps: First, the performance of a single feature was quantified by calculating the independent classification accuracy *Acc*(*s*, *k*, *f*_*i*_) of a feature *f*_*i*_ on the species *s* using a fivefold cross-validation strategy, which was calculated as follows:

(1) $$Acc(s,k,\;f_{i})=\frac{1}{N_{test}}\overset{N_{test}}{\underset{m=1}{\sum }}I(y_{test}^{\left(m\right)}={\hat{y}}_{test}^{\left(m\right)}),$$

where *I* is the indicator function, and *y*_*test*_, ${\hat{y}}_{test}$ represent the true label and the prediction result, respectively.

Second, generate feature utility ranking *R*(*s*, *k*) = [*f*_(1)_, *f*_(2)_, …, *f*_(*i*)_, …, *f*_(14)_] for pairs determined by species and model (*s*, *k*), where *f*_(*i*)_ denotes the optimal features for (*s*, *k*) pairs, in *Acc*(*s*, *k*, *f*_*i*_) descending order.

Through feature-model cross-evaluation, we establish a 3D decision space containing 6 × 3 × 14 evaluation units, thereby quantifying the difference of feature selectivity between different model architectures on different species data.

The second stage is dynamic feature fusion. In this stage, the incremental feature combination optimization is carried out based on the ranking results to realize the dual-adaptive mechanism. For each (*s*, *k*) pair, 14 candidate feature subsets ${\left\{F_{n}^{\left(s,k\right)}\right\}}_{n=1}^{14}$ are constructed in order *R*(*s*, *k*), where $F_{n}^{\left(s,k\right)}={⋃}_{i=1}^{n}\;f_{\left(i\right)}$ denotes the union of the previous *n* features. Based on model adaptation, the prediction performance $Acc(F_{n}^{\left(s,k\right)})$ of each $F_{n}^{\left(s,k\right)}$ is evaluated on the validation set, and the n* that maximizes the validation accuracy is selected:

(2) $$n^{*}=arg\underset{n\in [1,14]}{\max}Acc(F_{n}^{\left(s,k\right)}).$$

Based on species adaptability, we allow different species to obtain different n* values when using different models for feature encoding. For example, for the CNN model, the n* of *A. thaliana* is six, whereas that of *C. elegans* is nine. Likewise, there are significant differences in the optimal feature combinations selected by different models under the same species (Supplemental Table S7).

Finally, the following objective function was used as the species-model joint optimization objective:

(3) $$max\overset{6}{\underset{s=1}{\sum }}\overset{3}{\underset{k=1}{\sum }}Acc(F_{opt}^{\left(s,k\right)}),$$

where *S* represents six species, and *K* represents for three models. By joint optimization, the objectives selected by n* satisfies $F_{opt}^{\left(s,k\right)}=F_{n^{*}}^{\left(s,k\right)}$.

The above computational framework incorporates phylogenetic feature selectivity and architecture-specific feature optimization through its dual-adaptive mechanism. The selected optimal features are provided in Supplemental Table S7. From the perspective of species, the framework establishes a species-specific feature ranking *R*(*s*, *k*), which can capture the property differences of DNA sequences of different species. For example, experimental results show that *A. thaliana* showed maximal compatibility with physicochemical features (e.g., NCP, EIIP), whereas *G. subterraneus* demonstrated optimal performance with sequence composition features (e.g., CKSNAP, *k*-mer). For the model perspective, our proposed feature selection framework optimizes the feature processing ability of different DL base models. For example, in the experimental results, for *A. thaliana*, CNN and Bi-LSTM only select six out of 14 candidate encodings, whereas Transformer selects 13 candidate encodings. The optimal encodings of different base models show significant difference.

### Evaluation metrics

Comparative benchmarking with existing predictors was conducted through six evaluation metrics: accuracy (ACC), sensitivity (SN), specificity (SP), Matthews correlation coefficient (MCC), area under the curve (AUC), and F1-score. The above metrics are defined as follows:

(4) $$ACC=\frac{TP+TN}{TP+TN+FP+FN},$$

(5) $$SN=\frac{TP}{TP+FN},$$

(6) $$SP=\frac{TN}{TN+FP},$$

(7) $$F1\;Score=1-\frac{TP+TN}{2\times TP+FP+FN},$$

(8) $$MCC=\frac{TP\times TN-FP\times FN}{\sqrt{\left(TP+FP)\times (TP+FN)\times (TN+FP)\times (TN+FN\right)}},$$

where TP, TN, FP, and FN represent the number of true-positive, true-negative, false-positive, and false-negative samples, respectively. AUC is a commonly used metric to evaluate the performance of binary classifiers. The *x*-axis of the ROC curve is the false-positive rate (FPR), and the *y*-axis is the true-positive rate (TPR). AUC is the area enclosed by the ROC curve against the *x*-axis. Using these metrics and the framework described above, we proceeded to evaluate the performance of EnDeep4mC, as detailed in the Results section.

## Results

EnDeep4mC framework has been evaluated through a series of experiments designed to validate the efficacy of its core components: the DFS mechanism, the heterogeneous ensemble architecture, and its overall predictive performance. The results are presented as follows.

### DFS of EnDeep4mC

**Figure 2.**
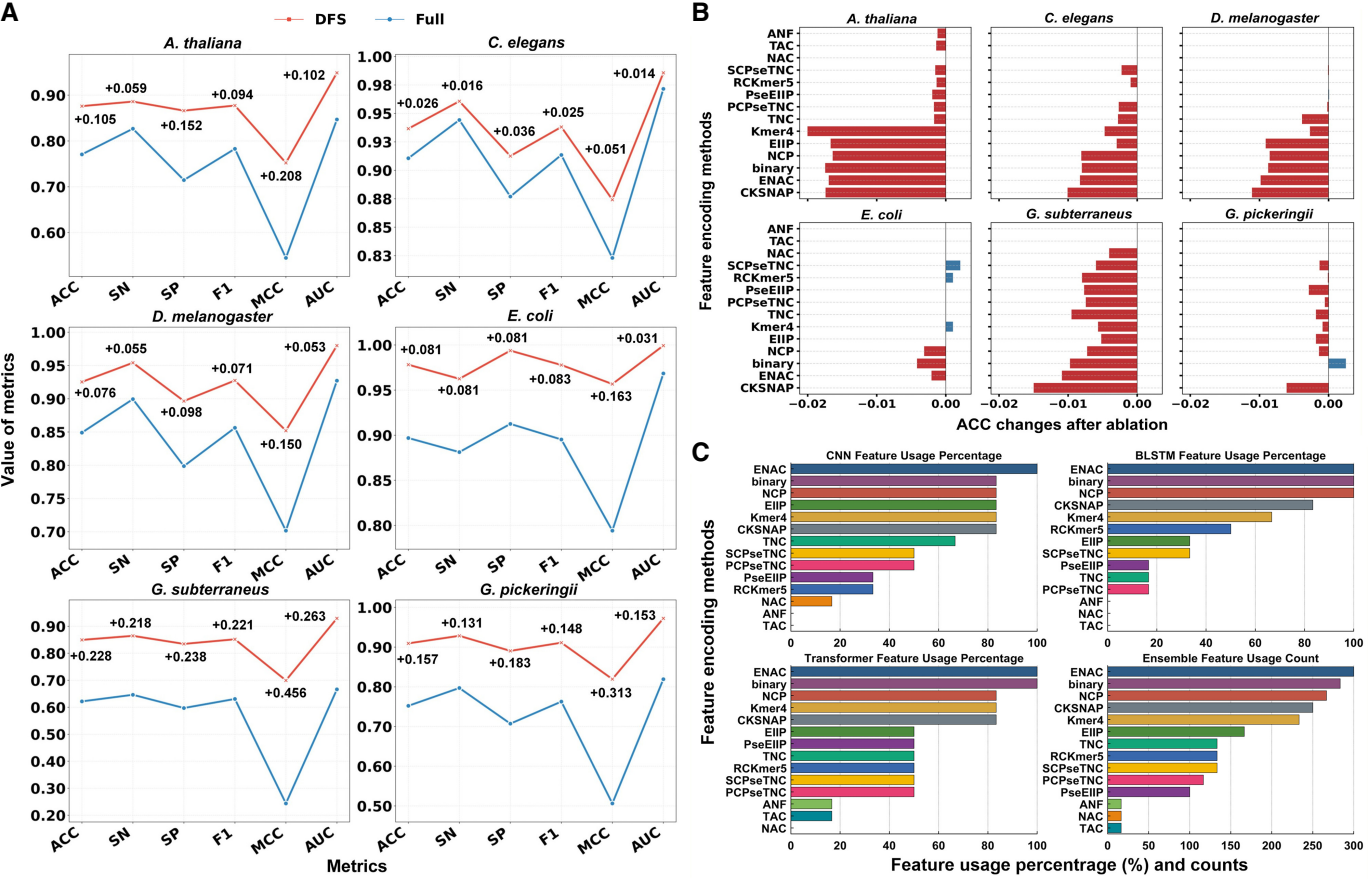
The impact of feature selection and encoding on EnDeep4mC's performance. (*A*) Metrics of the ensemble model using DFS versus full 14 encodings on the data sets of six species. (*B*) ACC changes of features selected by the DFS framework after ablation (Supplemental Tables S8–S10). (*C*) The selection rates of 14 feature encoding schemes on different base models and the total usage frequencies on ensemble model.

to validate the performance gains of the DFS framework, we conducted a systematic comparison against the baseline implementation using the 14 encoding schemes listed in Supplemental Table S6. As Figure 2A shows, the dual-adaptive DFS framework exhibited systematic performance gains on the data sets of six species. Specifically, the proposed framework improved the average AUC and ACC of the ensemble model by 11.84% (DFS: 0.9691 vs. full: 0.8666) and 14.05% (DFS: 91.25% vs. full: 80.01%), respectively. All the six metrics were positively improved in all species (+17.43% on average) (for feature selection details, Supplemental Fig. S3).

The results show that the feature selection framework can eliminate redundant and noisy features (such as TAC, ANF, etc.) while strengthening species-specific feature expression, as evidenced by NCP selectivity in *A. thaliana* and CKSNAP selectivity in *G. subterraneus*, through co-optimization of species-specific feature selectivity and model architectural characteristics. Collectively, these findings confirm that the proposed dual-adaptive framework enables the model to focus on the key discriminative features of 4mC sites, which significantly improves the prediction performance of EnDeep4mC.

To evaluate the impact of feature encoding schemes on EnDeep4mC, we conducted ablation experiments on the 14 candidate encoding schemes. We removed each feature selected in different DL base models through the DFS framework to examine the performance changes of the model on data sets of all six species. As Figure 2B shows, the average performance variation results of the cross-model analysis. The removal of 83.6% of the features led to a significant decline in model performance, with an average ACC decrease of 0.54%, demonstrating the efficacy of the DFS framework. Among them, features such as CKSNAP, ENAC, EIIP, and *k*-mer exhibited universal cross-species contributions. When these features were removed in cross-species scenarios, ACC decreased by an average of 0.55% to 1.19%. Particularly, the removal of *k*-mer resulted in an average decrease of 2.00% in ACC on the *A. thaliana* data set. Meanwhile, the *k*-mer encoding was also the one and only important encoding that ranked among the top six in all base models and all species. Notably, performance improvements were observed in *E. coli* upon removing SCPseTNC, RCK-mer, and *k*-mer, potentially owing to feature redundancy in small sample contexts.

Furthermore, model-specific effects were observed. In the *G. pickeringii* data set, ablating Binary encoding reduced CNN accuracy by 0.39% but increased Bi-LSTM accuracy by 0.80%, underscoring the architecture-dependent utility of encoding schems. These findings verify the necessity of the species-model collaborative optimization framework, which helps to mitigate adverse effects in heterogeneous models.

We also systematically analyzed the feature selection patterns of the DFS framework across all base models (Fig. 2C). ENAC, Binary, and NCP exhibited dominance, with average selection rates of 100%, 94.44%, and 88.89%, respectively. Especially, ENAC achieved the maximum theoretical selection frequency (18 instances), highlighting its universal representational ability for cross-species 4mC prediction. In contrast, encoding schemes such as ANF, NAC, and TAC were not selected in most species, suggesting that their representational capabilities might be limited by specific sequence context environments.

### Comparison with single base models

In this section, we further evaluated the performance comparison of EnDeep4mC and its three base models with fivefold cross-validation on different species data sets, with key metrics reported as mean ± standard deviation. The narrow 95% confidence intervals (typically within ±0.5–1.5% of the mean) confirm the stability of the results across different data partitions. As summarized in Table 1 and detailed in Supplemental Tables S11 through S16 and Supplemental Figure S4, the EnDeep4mC ensemble outperformed the other individual models across all species, with average ACC and AUC of 94.99% and 0.9859, respectively, representing 4.52% and 2.12% improvements over the best base model (Transformer). Notably, EnDeep4mC achieved near-optimal performance on *E. coli*, with an ACC of 99.71% and an AUC of 0.9999, approaching theoretical perfection.

**Table 1.** Performance comparison between EnDeep4mC ensemble model and each base model trained by fivefold cross-validation on six species data sets

Data set	Algorithm	ACC	SN	SP	MCC	AUC	F1-score
*C. elegans*	CNN	0.9252	0.9559	0.8946	0.8529	0.9812	0.9278
	Bi-LSTM	0.9231	0.9514	0.8948	0.8461	0.9792	0.9246
	Transformer	0.9296	0.9589	0.9003	0.859	0.9829	0.9308
	EnDeep4mC	**0.9571**	**0.9594**	**0.9548**	**0.9142**	**0.9914**	**0.9572**
*D. melanogaster*	CNN	0.9191	0.9530	0.8851	0.8401	0.9758	0.9216
	Bi-LSTM	0.9169	0.9502	0.8836	0.8379	0.9738	0.9205
	Transformer	0.9246	**0.9540**	0.8951	0.8512	0.9778	0.927
	EnDeep4mC	**0.9412**	0.9508	**0.9316**	**0.8826**	**0.9842**	**0.9418**
*A. thaliana*	CNN	0.8727	0.8933	0.8522	0.7489	0.9453	0.8758
	Bi-LSTM	0.8702	0.8781	0.8623	0.7423	0.9423	0.8727
	Transformer	0.8789	0.8962	0.8617	0.7587	0.9491	0.8811
	EnDeep4mC	**0.9133**	**0.9205**	**0.9061**	**0.8267**	**0.9697**	**0.9133**
*E. coli*	CNN	0.9619	0.9513	0.9725	0.9438	0.9933	0.9718
	Bi-LSTM	0.9644	0.9437	0.9850	0.9251	0.9972	0.9623
	Transformer	0.9675	0.9613	0.9737	0.9376	0.9947	0.9686
	EnDeep4mC	**0.9973**	**0.9969**	**0.9976**	**0.9945**	**0.9999**	**0.9973**
*G. subterraneus*	CNN	0.8525	0.8725	0.8325	0.7102	0.9334	0.852
	Bi-LSTM	0.8551	0.8545	0.8557	0.7142	0.9309	0.8578
	Transformer	0.8514	0.8527	0.8500	0.7024	0.9282	0.8513
	EnDeep4mC	**0.9349**	**0.9323**	**0.9375**	**0.8698**	**0.9786**	**0.9347**
*G. pickeringii*	CNN	0.9075	0.9437	0.8712	0.8233	0.9722	0.9138
	Bi-LSTM	0.9180	0.9384	0.8976	0.8345	0.9742	0.9186
	Transformer	0.9107	0.9346	0.8868	0.8241	0.9709	0.9127
	EnDeep4mC	**0.9729**	**0.9740**	**0.9718**	**0.9457**	**0.9937**	**0.9729**

Bold values indicate the best performance achieved within each comparison group.

Among the base models, CNN and Bi-LSTM showed comparable performance. The Transformer was demonstrated the strongest overall performance (average ACC = 90.47%), outperforming CNN (90.30%) and Bi-LSTM (90.45%). It also exhibited stable performance on *C. elegans* and *D. melanogaster*, with ACC reaching 92.96% and 92.46%, respectively. Notably, Transformer achieved superior sensitivity (SN = 0.9545) on *D. melanogaster*, even surpassing EnDeep4mC by 0.0037, which might be because of the stronger ability of Transformer to capture the global features of specific sequential patterns.

Through cross-species comparative analysis, it was also found that the standard deviation of ACC of EnDeep4mC was 3.62%, which was lower than that of the base models (3.85%–4.12%), demonstrating that its ensemble strategy effectively mitigated data bias sensitivity. In addition, the AUC of EnDeep4mC in *A. thaliana* and *G. pickeringii* reached 0.9697 and 0.9999, respectively, which further verified its generalization ability. The performance improvements were particularly significant on microbial species: EnDeep4mC achieved 7.98% and 5.49% ACC gains over the best base model on *G. subterraneus* and *G. pickeringii*, respectively, highlighting the framework's robustness in handling phylogenetically diverse data sets. Therefore, the EnDeep4mC framework is feasible on all benchmark data sets of DNA 4mC, as well as its superiority over traditional single DL models in terms of prediction accuracy, robustness, and cross-species adaptability.

### Ablation experiment of EnDeep4mC model structure

**Figure 3.**
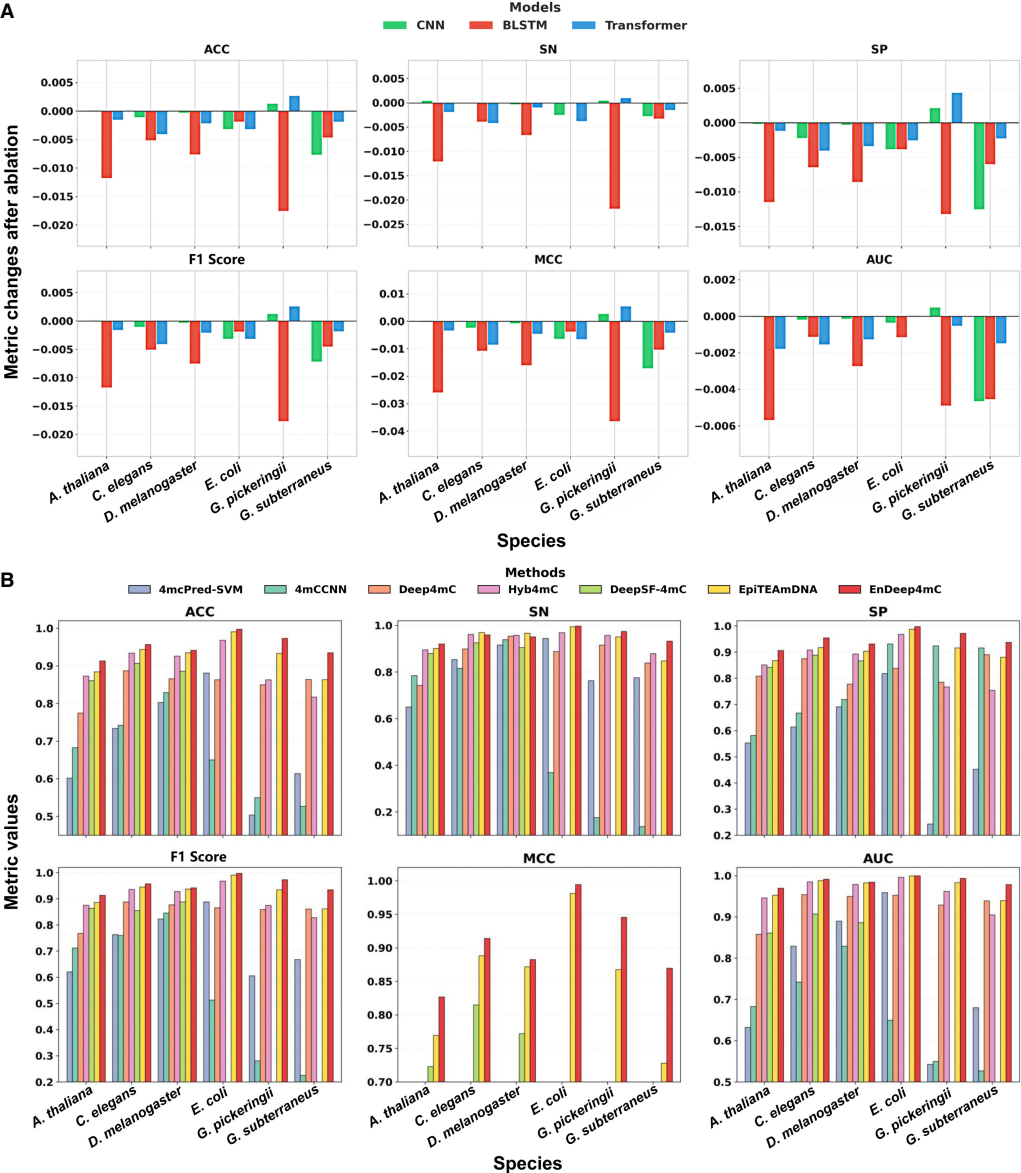
Comparative performance analysis of 4mC prediction methods. (*A*) Metrics changes of the system after removing each base model, respectively, in the EnDeep4mC framework (Supplemental Tables S17–S19). The vertical axis represents the relative change values of each metric. (*B*) Comprehensive performance comparison of seven 4mC prediction methods across multiple species and evaluation metrics. Blank values indicate that the corresponding metric was not reported in the original publication.

The probabilistic fusion architecture of EnDeep4mC relies on the complementary integration of CNN, Bi-LSTM, and Transformer. To validate their synergistic effects, we systematically excluded each single base model and retrain the ensemble system to quantify the performance change on independent test sets of six species (Fig. 3). The results demonstrated that the removal of any single base model consistently degraded the ensemble's performance, and the decrease effect is species specific. As shown in Figure 3, excluding the Bi-LSTM had the largest negative impact on average ACC (−0.78%), AUC (−0.34%), and SP (−0.58%), with a notable effect on microbial groups such as *G. subterraneus* (−0.43% ACC). The removal of Transformer significantly impacted the average MCC (−0.56%) and F1-score (−0.39%), especially in animal groups (e.g., *C. elegans* with −0.79% in MCC). Notably, excluding CNN and Transformer from the ensemble surprisingly slightly improved ACC in *G. pickeringii*, potentially owing to their local-global feature redundancy.

The analysis above highlight the complementary roles of the three base models. CNN provided the most stable performance for prokaryotes owing to its ability to parse short-range local patterns. Bi-LSTM effectively captured the sequence dependence of negative samples through bidirectional time series modeling, and its removal led to an average reduction of 0.58% in specificity (SP). The Transformer relied on the self-attention mechanism to strengthen the global correlation discrimination, which significantly improved the prediction accuracy of class imbalance samples. In summary, by integrating these heterogeneous base models, EnDeep4mC dynamically adapted to multiscale sequence features, providing actionable insights into ensemble architecture design in DL frameworks.

### Comparison with existing predictors

To verify the performance of EnDeep4mC, we systematically compared it against five DL models (4mCCNN, Deep4mC, Hyb4mC, DeepSF-4mC, and EpiTEAmDNA) and one traditional ML model (4mcPred-SVM). To ensure a fair and reproducible comparison with existing predictors, our benchmarking analysis adhered to the following principles: All models were evaluated on the identical benchmark data sets curated from EpiTEAmDNA, employing the same fivefold cross-validation splits and evaluation metrics. Comprehensive benchmarking analyses (Fig. 3B) demonstrate that EnDeep4mC's significant outperformance across key evaluation metrics. EnDeep4mC outperformed all baselines with average ACC of 95.28% and AUC of 0.9863, surpassing the suboptimal model EpiTEAmDNA by 2.76% (ACC) and 1.21% (AUC). Notably, EnDeep4mC achieved exceptional performance on *E. coli* (ACC = 99.73%, AUC = 0.9999), outperforming EpiTEAmDNA by 0.67% in ACC. This represents one of the highest reported accuracy rates, surpassing 90% across all benchmark species.

To ensure a statistically rigorous comparison, we conducted a side-by-side evaluation of EnDeep4mC against two prominent ensemble-based baselines—Hyb4mC and the current state-of-the-art EpiTEAmDNA—under consistent experimental conditions (identical data sets, fivefold CV). As detailed in Supplemental Table S20, EnDeep4mC achieves consistent and often substantial improvements across all six species. For instance, it attains a notable AUC gain of 11.2% over Hyb4mC on *G. subterraneus*, while maintaining a stable advantage over EpiTEAmDNA even on high-performing data sets like that of *E. coli*. These results validate the effectiveness of the species-model collaborative feature selection strategy, which enhances the prediction accuracy and robustness by adaptively fusing local conservation (CNN), long-range dependence (Bi-LSTM), and global correlation features (Transformer).

To assess the compatibility of EnDeep4mC with emerging detection platforms, we also evaluated the framework on independent data derived from a latest Oxford Nanopore Technologies (ONT) sequencing study (Galeone et al. 2025). Using motif-based labeling of 4mC and non-4mC sites from *Enterococcus faecium*, *Klebsiella pneumoniae*, and *Listeria monocytogenes*, we constructed a FASTA-format data set of variable length (Supplemental Table S21). EnDeep4mC and its base models all delivered robust performance on this set (Supplemental Table S22), with the ensemble attaining an average AUC more than 0.9999 across the three species. These results indicate that our sequence-based predictor retains competitive accuracy even when applied to data generated by state-of-the-art ONT sequencing, highlighting its potential as a rapid, cost-efficient screening tool that can usefully complement experimental profiling efforts.

### Cross-predictions between different species

**Figure 4.**
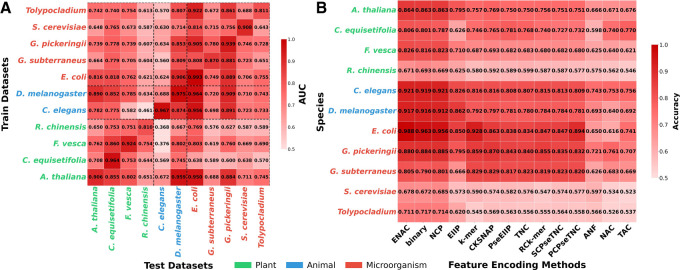
Prediction performance on different species groups, in which species names are green for plant group, blue for animal group, and red for microbial group. (*A*) Cross-species prediction performance (AUC) evaluated on 11 species, including held-out subsets from the six benchmark species to assess zero-shot transfer to previously unseen data. (*B*) Prediction performance on independent test data sets of 11 species using 14 feature encodings respectively, taking CNN as an example (Supplemental Fig. S6).

To evaluate the cross-species generalization capability of EnDeep4mC, we performed systematic prediction across a broader range of species. The cross-prediction performance was visualized in a heatmap using AUC and ACC as indicators (Fig. 4A; Supplemental Fig. S5), which revealed the differences in the transfer ability. Notably, we adopted a strict feature inheritance protocol: For zero-shot transfer, target species sequences were encoded exclusively using the feature encoding scheme optimized for the source species during its training.

As shown in Figure 4A, the 4mC data sets from EpiTEAmDNA were categorized into plant, animal, and microbial groups. Cross-prediction results showed that the diagonal species achieved the highest performance (average ACC = 83.36%, AUC = 0.9138), validating intraspecies transfer reliability. Among them, *D. melanogaster* exhibited the best cross-species generalization ability in our model, achieving AUC of 0.9639 for *E. coli* and 0.9090 for *G. pickeringii*. In contrast, cross-group predictions were markedly less accurate. For instance, plant-to-microbial transfers showed an average AUC of 0.6851, a 25.0% reduction compared to intraspecies predictions.

Notably, *Fragaria vesca* achieved an AUC of 0.8028 in predicting *E. coli*—4.3% higher than intragroup microbial transfer (0.7696), indicating conserved methylation features in its sequences. The performance of intramicrobial prediction outperformed other groups with an average AUC of 0.7696, with *Tolypocladium*–to–*E. coli* prediction achieving AUC = 0.9216, which might be related to the homology of the modification system in prokaryotes.

These observed performance patterns, strong intragroup predictability alongside attenuated cross-group transfer, directly support the utility of more targeted training strategies. Our results indicate that models trained specifically for a particular biological group (e.g., prokaryotes or plants) are likely to achieve superior performance when predicting 4mC sites in newly sequenced genomes from phylogenetically related organisms, compared with a single universal model (Zemach et al. 2010; Beaulaurier et al. 2019).

Furthermore, we identified specific species as acting as “knowledge hubs.” The model trained on *D. melanogaster* achieved AUC >0.7 in 80% cross-predictions and exhibited superior global transfer stability (standard deviation = 0.1269) compared with other species (average standard deviation = 0.1526). This property could be related to the global methylation pattern of DNA, and its sequences might capture more universal features of 4mC modification. These findings imply that integrating feature encoding schemes from multiple species is expected to further improve the prediction stability of the model in cross-group species.

### Selectivity of different species for feature encoding schemes

Species-specific selectivity for feature encoding schemes was further analyzed across 11 species. We encoded all the 11 data sets from EpiTEAmDNA by 14 feature encoding schemes and evaluated ACC via three DL base models on independent test sets, thus quantifying feature selectivity across species and models to uncover encoding specificity. For cross-group analysis, 11 species were still categorized into three groups: plants, animals, and microbes.

The results revealed significant feature encoding selectivity across three biological groups (as shown in Fig. 4B; Supplemental Fig. S6). Plant species showed optimal performance with global statistical encodings: *A. thaliana* achieved 86.39% ACC with ENAC, whereas *F. vesca* attained 82.58% with the same encoding. Local sequential patterns like CKSNAP showed moderate performance (average ACC = 70.48% across four plant species), with *Casuarina equisetifolia* reaching 76.50% ACC, indicating plants’ preference for global sequence features (Zilberman et al. 2007).

Animal species exhibited strong preference for nucleotide chemical property (NCP) and global encodings. *C. elegans* achieved 92.11% ACC with NCP, whereas *D. melanogaster* attained 91.66% with ENAC. Notably, NCP encoding outperformed local *k*-mer encoding by 10.19% in *C. elegans*, suggesting animals’ reliance on nucleotide-level chemical features for methylation recognition (Feng et al. 2010).

Microbial groups displayed significant prokaryotic-eukaryotic differentiation. Prokaryotes (*E. coli*, *G. subterraneus*, *G. pickeringii*) excelled with both global and local encodings: *E. coli* achieved exceptional 98.75% ACC with ENAC, whereas *G. pickeringii* attained 85.85% with *k*-mer. Eukaryotes (*S. cerevisiae*, *Tolypocladium*) showed lower performance across all encodings, with *S. cerevisiae* achieving only 67.80% ACC via NCP, 22.95% below the prokaryotic average (90.73%), reflecting sequence heterogeneity in eukaryotic epigenetic regulation (Jones 2012).

In addition, group stability analysis revealed animals exhibited the lowest coefficient of variation (CV = 0.094), whereas plants (CV = 0.120) and microbes (CV = 0.200) showed higher volatility, consistent with feature granularity differences. These findings provide optimization guidelines for species-feature adaptation: Plant groups benefit from global statistical encodings (ENAC, binary); animal groups perform best with NCP encodings; and microbial groups require differentiation between prokaryotic and eukaryotic encoding schemes.

These findings provide a new perspective for optimization of species-feature adaptation. On the whole, plant groups show a preference for global statistical encodings (e.g., ENAC, binary) rather than local patterns, suggesting global sequence composition is more critical for methylation recognition in plants. Animal groups consistently perform best on NCP encodings and global statistical features. Microbial groups maintain significant prokaryotic–eukaryotic differentiation, necessitating distinct encoding strategies for prokaryotic versus eukaryotic species.

## Discussion

EnDeep4mC achieves state-of-the-art prediction performance and provides novel insights into the evolutionary dynamics of DNA 4mC modification. A key insight is its enhanced intraclass transferability for prokaryotes, as evidenced in cross-species predictions. These observations are consistent with the hypothesis that this stems from the homology of their modification systems. However, some eukaryotic species exhibited global transfer stability, possibly reflecting their global DNA methylation pattern. Furthermore, cross-group *k*-mer spectrum analysis (Supplemental Methods) revealed an evolutionary divergence: Prokaryotes maintain the functional stability of the RM system through conserved short *k*-mers, whereas eukaryotes achieve epigenetic plasticity through diverse sequence combinations. Additionally, Supplemental Figure S7 shows vertically compact, horizontally dispersed warm-colored regions, revealing two evolutionary insights. First, multiple significant *k*-mers at a specific *k*-value indicate the strong selection pressure at that motif length. For example, the proportion of warm colors at *k* = 3 was higher than that at other *k*-values, implying that the scale conservation of 4mC sequences was strong under 3-mer. Second, fluctuations in significance levels as *k*-values vary reveal length-dependent selection. For the *k*-mer sequences of “AAA,” the significance level gradually decreased when the *k*-value increased from three to five, indicating the specificity of 4mC sequences in terms of length.

We next examined *k*-mer enrichment differences between prokaryotes and eukaryotes (Supplemental Fig. S8). At the *k* = 3 scale, prokaryotes exhibited highly specific *k*-mer enrichment, with motifs such as “GGC” and “CGG” showing significant positive differences. The strong conservation of these motifs in prokaryota suggests that their 4mC modification system may be under strict coevolutionary selection pressure to ensure the exact match between methylation sites and restriction sites in their host RM system (Vasu and Nagaraja 2013). This finding aligns with the observed intraclass transferability advantage, suggesting that conserved sequence features could enhance the cross-species prediction generalizability.

Unlike prokaryotes, eukaryotes showed dynamic sequence selectivity across different *k*-values. For example, in Supplemental Figure S7, the enrichment intensity of “AAA” showed a gradient decline with the increase of *k*-value, suggesting that eukaryotes may achieve the improvement of regulatory accuracy through the expansion of sequence length. Notably, most of the significant *k*-mers showed a bidirectional distribution (e.g., “CGG” was enriched in prokaryotic but absent in eukaryotic at *k* = 3). This polymorphism may reflect the adaptive requirement of 3D chromatin structure (Zhou et al. 2019) and could indicate that regions rich in repetitive sequences (such as poly(A)) are more likely to form open chromatin conformations, thus facilitating the accessibility of methyltransferases (Schübeler 2015).

Frequency difference polarization revealed a clear functional divergence between prokaryotes and eukaryotes: 83.3% of significant *k*-mers in prokaryotes showed unidirectional enrichment, whereas 93.3% of significant *k*-mers in eukaryotes showed background selectivity. These patterns are consistent with the hypothesis of different evolutionary selection pressures. Prokaryotes appear to be subject to the functional constraints of the host RM system to maintain the sequence conservation of methylation sites, whereas eukaryotes, potentially driven by developmental regulation, may dynamically modify heterogeneous DNA regions (such as gene spacer regions) to achieve spatiotemporal-specific epigenetic encoding (Suzuki and Bird 2008). Furthermore, *k*-mers frequently found in eukaryotic negative samples (e.g., “CCC,” “GGG,” “GCC,” etc.) potentially act as an “antimethylation” signal, analogous to the methylation suppression phenomenon of CpG islands in mammals (Deaton and Bird 2011).

Despite the robust cross-species predictive performance of EnDeep4mC, our approach is subject to certain limitations. Specifically, the inference of methylation profiles across species inherently assumes conserved regulatory mechanisms mediated by methyltransferases. In prokaryotes, in which methylation is often governed by RM systems with highly specific sequence motifs, the absence of prior knowledge regarding the active methyltransferase repertoire in a target species may constrain prediction accuracy. Our cross-species validation, while demonstrating promising transferability within microbial groups, also underscores the necessity of phylogenetic context when applying the model to distantly related organisms. For instance, although *E. coli* and *G. pickeringii* (both prokaryotes) exhibit strong intragroup predictability, extrapolation to uncharacterized prokaryotes without methyltransferase annotation remains challenging.

Looking forward, as genomic and epigenomic data sets continue to expand, covering increasingly diverse species and methylation systems, we anticipate that models like EnDeep4mC will gain further robustness and biological interpretability. The integration of methyltransferase annotation, structural motifs, and evolutionary context into future frameworks will be crucial to systematically explore the variation of modification patterns across species, ultimately uncovering deeper mechanisms of epigenetic evolution.

## Code availability

An integrated web server combining the ensemble framework and DFS module was implemented for public access at http://lab.malab.cn/~lxm/EnDeep4mC. The platform supports species selection and accepts DNA 4mC sequence inputs either through FASTA-formatted text entry or through file upload. Prediction results are generated as interactive web reports or downloadable text documents, providing systematic analysis of 4mC modifications. The server architecture ensures user-friendly operation while maintaining the computational accuracy demonstrated in our methodology. The source code and data sets are publicly available at GitHub (https://github.com/RaySYZhang/EnDeep4mC) (release v1.0), with detailed implementation and usage described in the Supplemental Methods. EnDeep4mC source code is also available as Supplemental Code.

## Supplemental Material

Supplement 1

Supplement 2
