# Supplementary figures and images for: EnDeep4mC predicts DNA N4-methylcytosine sites using a dual-adaptive feature encoding framework in deep ensembles
Source: Genome Res. 2026 Mar;36(3):589–99. doi: 10.1101/gr.280977.125 (PMC12951953; doi:10.1101/gr.280977.125)

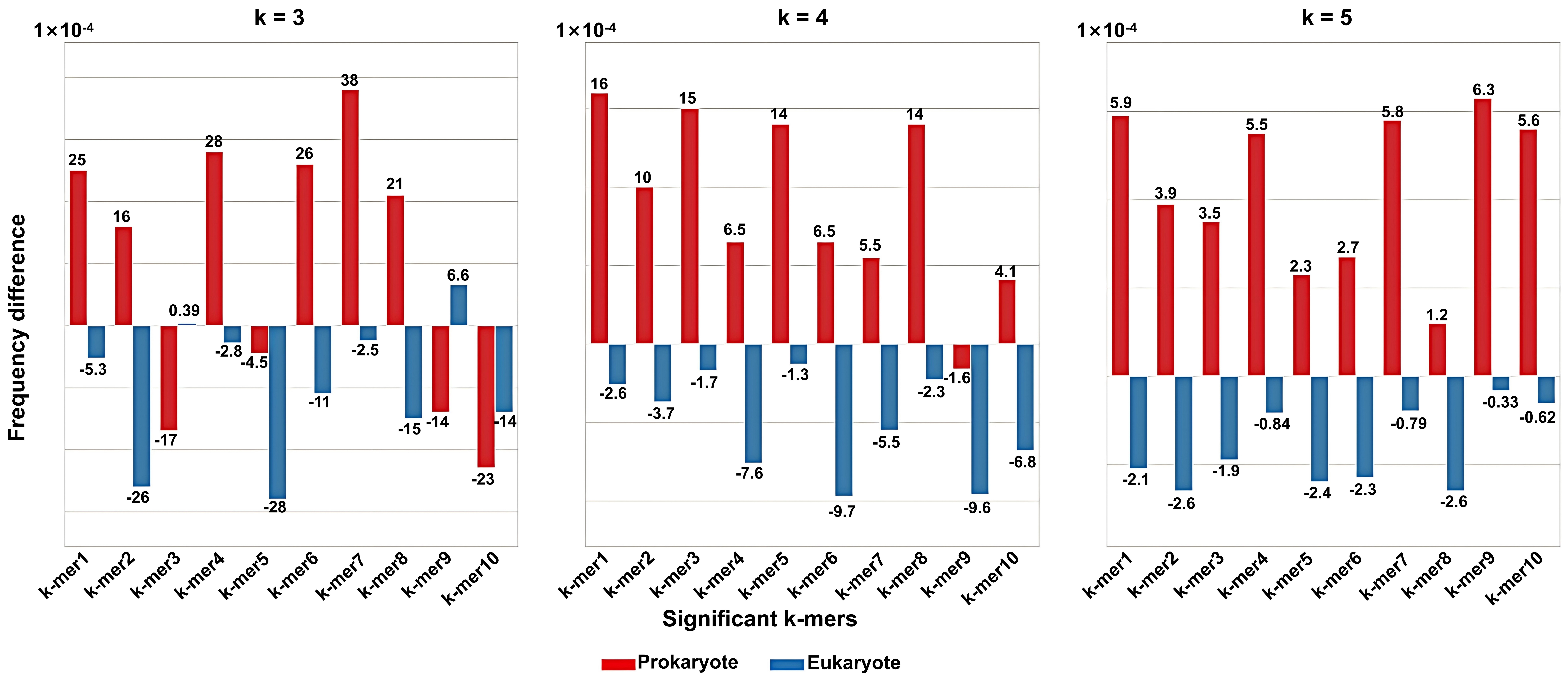

Supplement: Supplement 1 [file Supplemental_Code.zip › Supplemental_Code/EnDeep4mC-main/evaluations/kmer_analysis/figures/k-mer frequency differences.jpg]

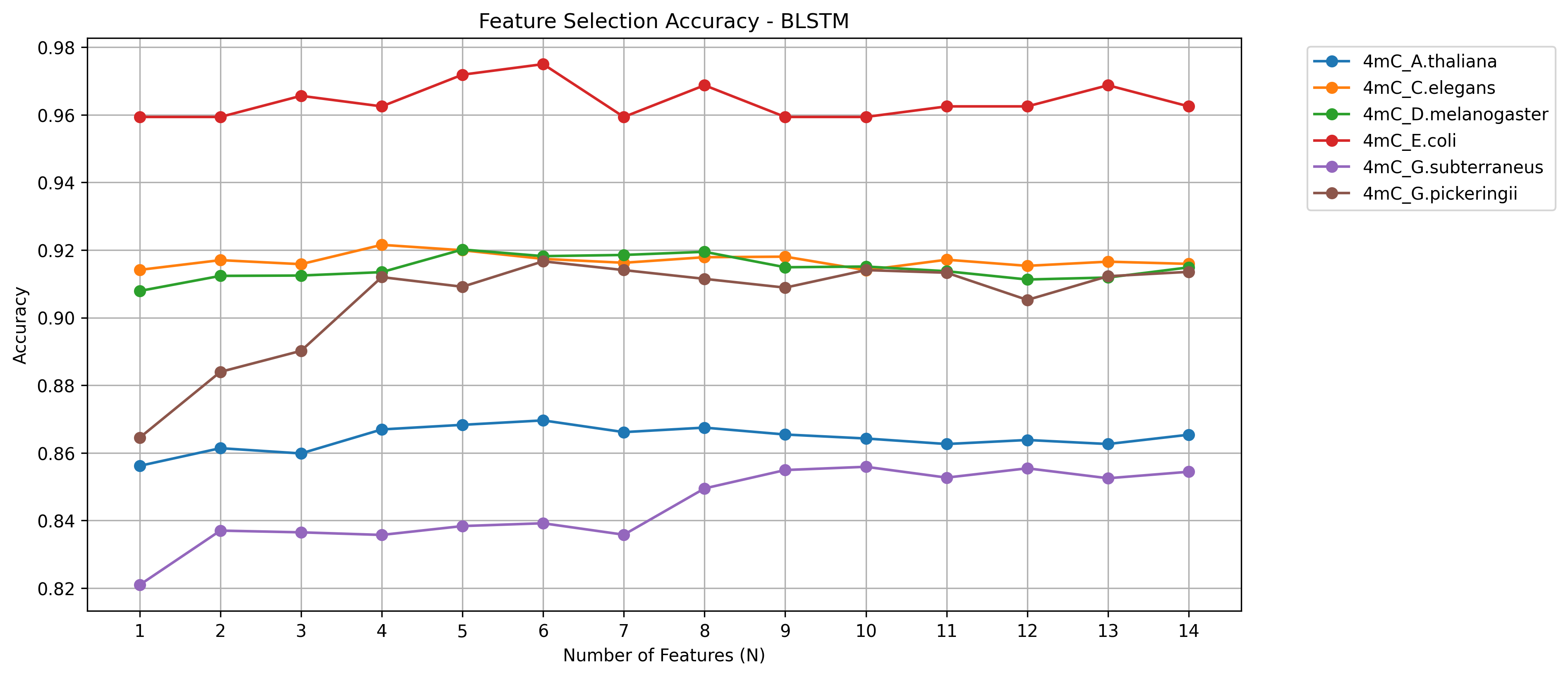

Supplement: Supplement 1 [file Supplemental_Code.zip › Supplemental_Code/EnDeep4mC-main/feature_engineering/ifs_result/Acc_Curves/BLSTM_Feature_Acc_Curve.png]

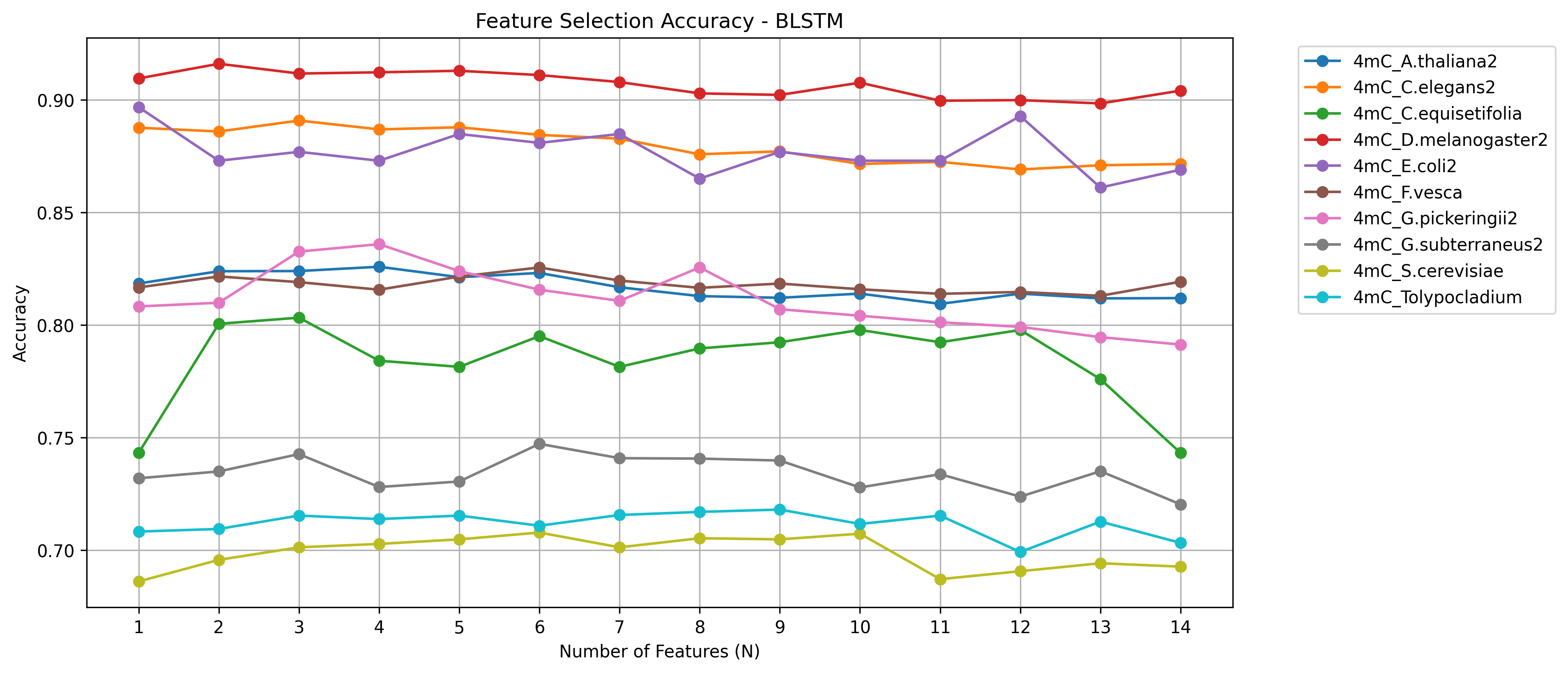

Supplement: Supplement 1 [file Supplemental_Code.zip › Supplemental_Code/EnDeep4mC-main/feature_engineering/ifs_result/Acc_Curves/BLSTM_Feature_Acc_Curve_extra_species.png]

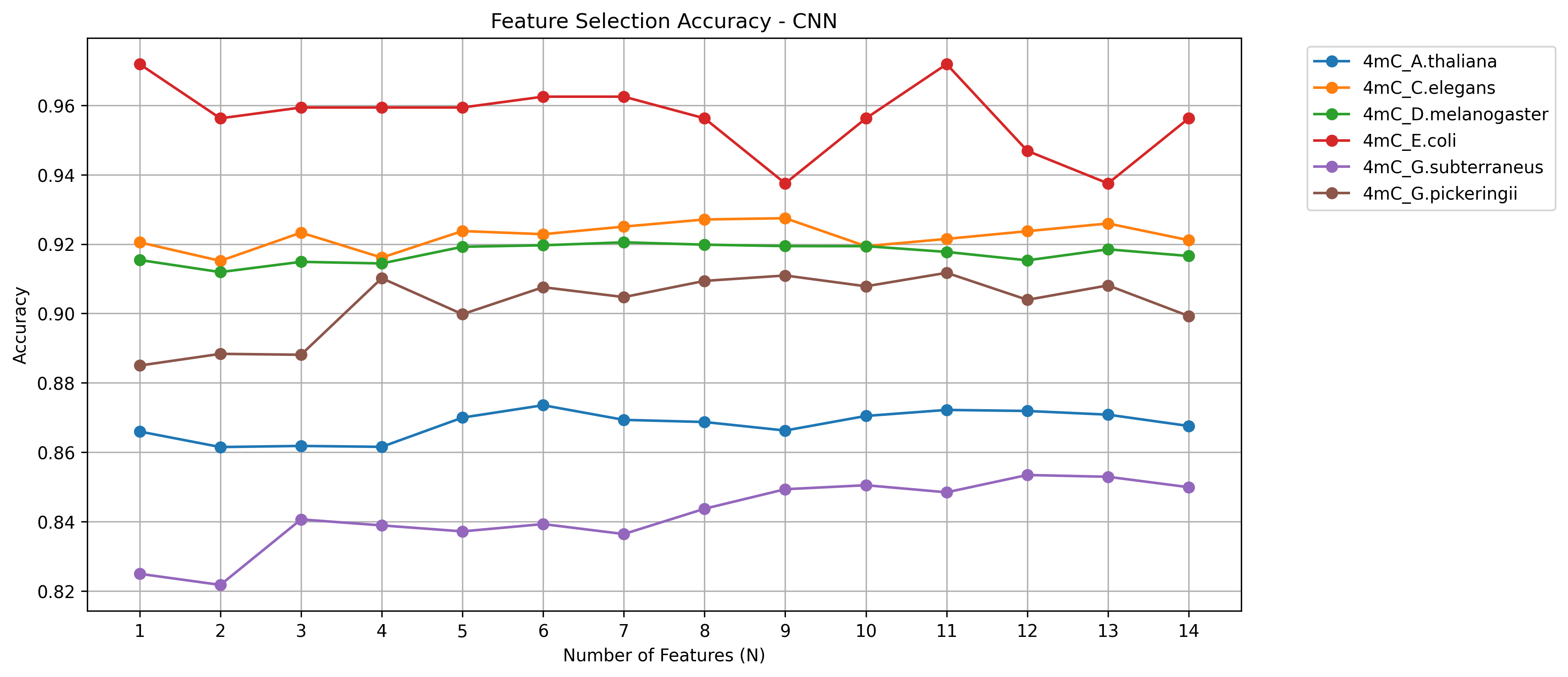

Supplement: Supplement 1 [file Supplemental_Code.zip › Supplemental_Code/EnDeep4mC-main/feature_engineering/ifs_result/Acc_Curves/CNN_Feature_Acc_Curve.png]

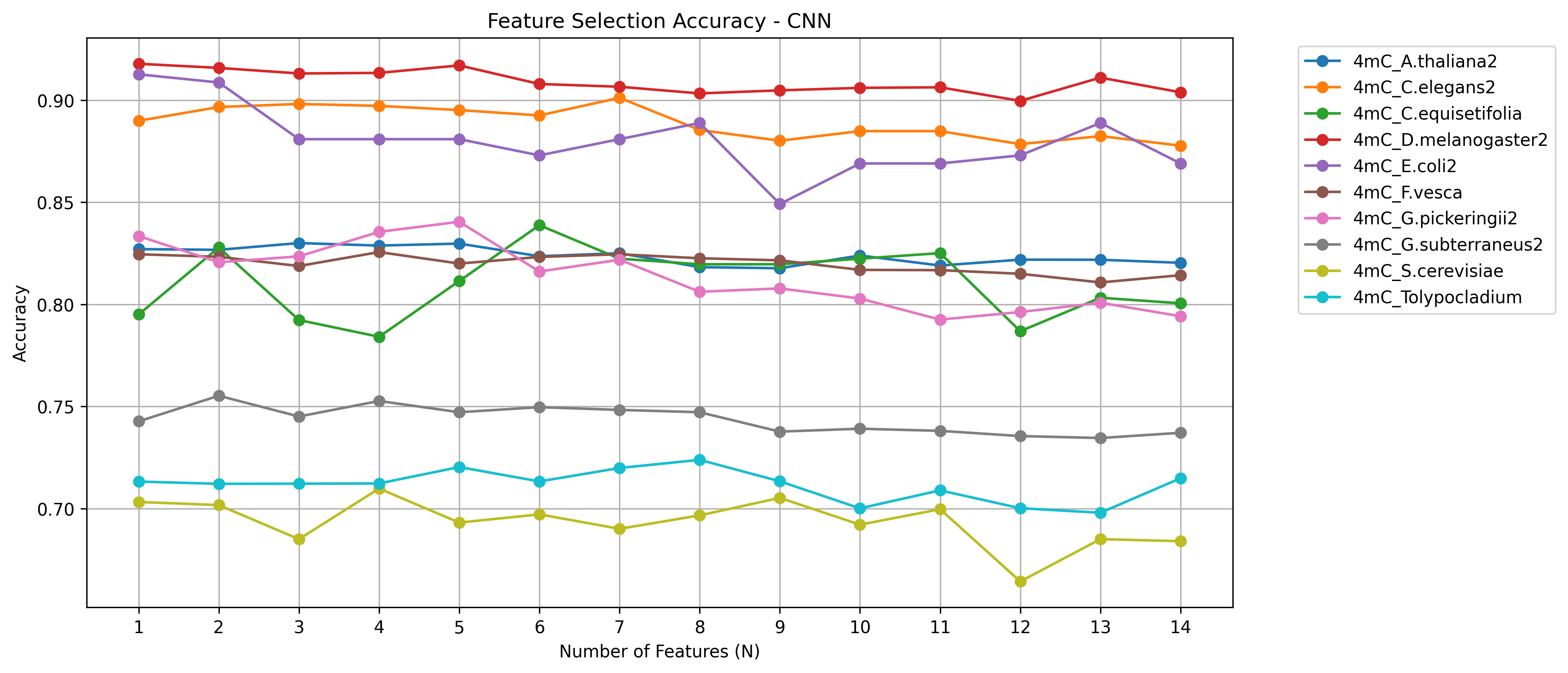

Supplement: Supplement 1 [file Supplemental_Code.zip › Supplemental_Code/EnDeep4mC-main/feature_engineering/ifs_result/Acc_Curves/CNN_Feature_Acc_Curve_extra_species.png]

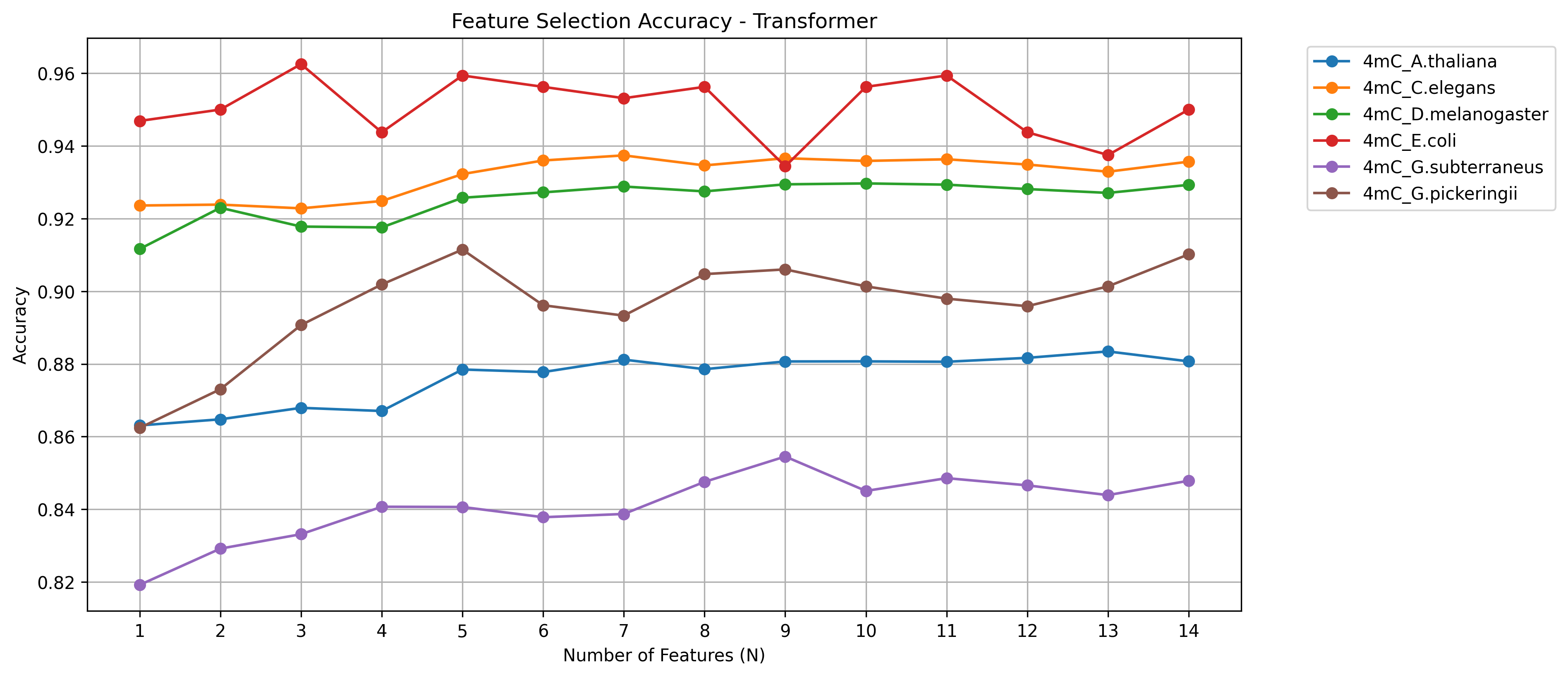

Supplement: Supplement 1 [file Supplemental_Code.zip › Supplemental_Code/EnDeep4mC-main/feature_engineering/ifs_result/Acc_Curves/Transformer_Feature_Acc_Curve.png]

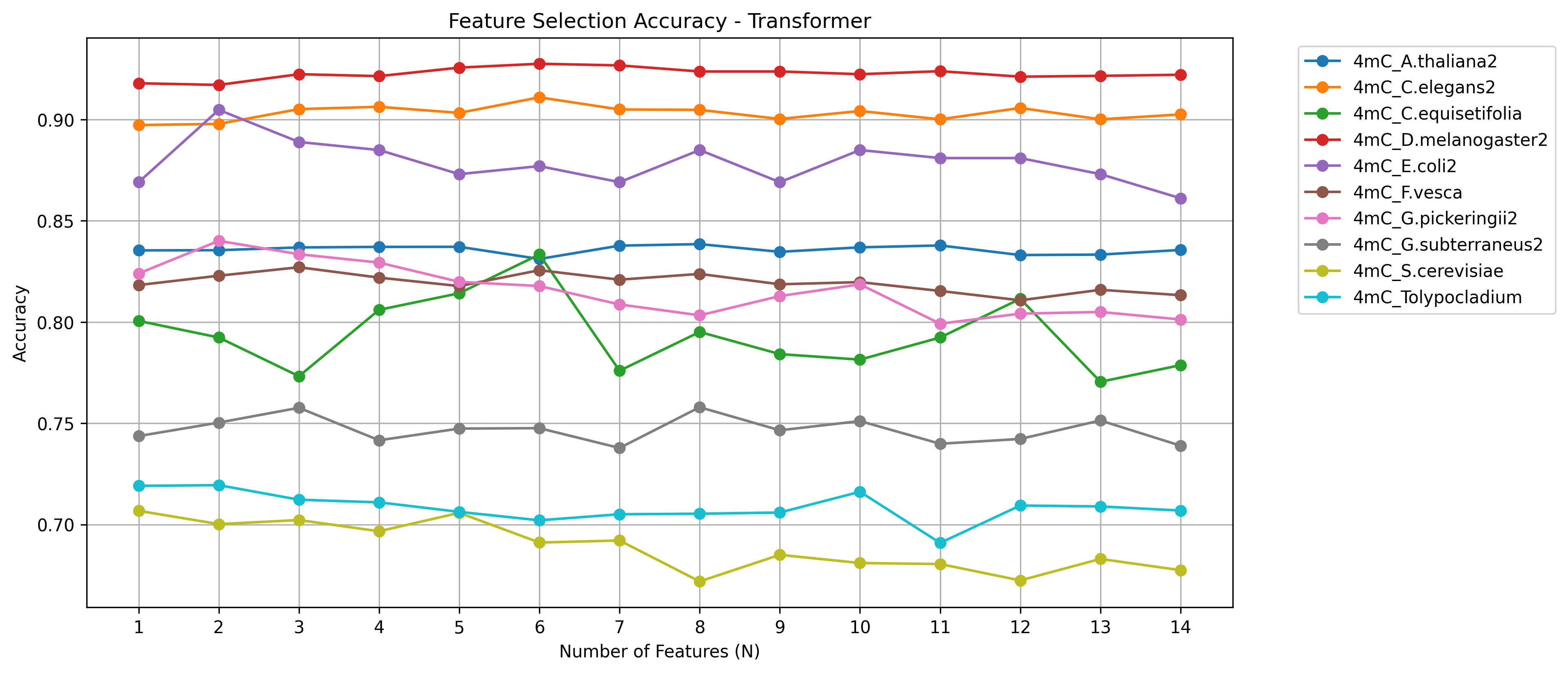

Supplement: Supplement 1 [file Supplemental_Code.zip › Supplemental_Code/EnDeep4mC-main/feature_engineering/ifs_result/Acc_Curves/Transformer_Feature_Acc_Curve_extra_species.png]
