# Supplementary material for: EnDeep4mC predicts DNA N4-methylcytosine sites using a dual-adaptive feature encoding framework in deep ensembles
Source: Genome Res. 2026 Mar;36(3):589–99. doi: 10.1101/gr.280977.125 (PMC12951953; doi:10.1101/gr.280977.125)
Supplement: Supplement 2 [file Supplemental_Materials.pdf]

Supplemental Material:

Supplemental Figure S1.Processing Pipeline for Variable-Length Sequences......1
Supplemental Figure S2.The t-SNE visualization of feature distributions of six species after data processing......2
Supplemental Figure S3. The distribution of the best feature sets of three base models on benchmark datasets of six species......3
Supplemental Figure S4. ROC curves of training the deep models on the benchmark datasets of six species......4
Supplemental Figure S5. Cross-prediction on datasets of 10 species using ACC as indicator......5
Supplemental Figure S6. Prediction performance on independent test sets of all 16 species after encoding by 14 feature encoding methods respectively......6
Supplemental Figure S7. The significance level distribution heatmap of k-mer at different k values in the k-mer analysis experiment......7
Supplemental Figure S8. Bar charts of the Top 10 significant k-mer frequency differences among eukaryotic/prokaryotic groups in the k-mer analysis experiment......8
Supplemental Table S1. Data Statistics of the Benchmark Datasets for 4mC Site Prediction Across Multiple Species......9
Supplemental Table S2.Diversity analysis for base models......10
Supplemental Table S3. Architecture and Hyperparameters of Base Deep Learning Models......10
Supplemental Table S4. Configuration of Ensemble Learning Framework......11
Supplemental Table S5. Training Configuration Parameters......11
Supplemental Table S6. Summary of the candidate feature encoding schemes in this paper......11
Supplemental Table S7. Feature sets of the benchmark datasets of 6 species selected by the dynamic feature selection (DFS) framework......12
Supplemental Table S8. The relative accuracy changes of the CNN model after ablating each feature selected by the DFS framework......13
Supplemental Table S9. The relative accuracy changes of the Bi-LSTM model after ablating each feature selected by the DFS framework......14
Supplemental Table S10. The relative accuracy changes of the Transformer model after ablating each feature selected by the DFS framework......14

Supplemental Table S11. Statistical performance analysis of models on *A. thaliana* (with 95% Confidence Intervals)....16
Supplemental Table S12. Statistical performance analysis of models on *C. elegans* (with 95% Confidence Intervals)....17
Supplemental Table S13. Statistical performance analysis of models on *D. melanogaster* (with 95% Confidence Intervals)....18
Supplemental Table S14. Statistical performance analysis of models on *E. coli* (with 95% Confidence Intervals)....19
Supplemental Table S15. Statistical performance analysis of models on *G. subterraneus* (with 95% Confidence Intervals)....20
Supplemental Table S16. Statistical performance analysis of models on *G. pickeringii* (with 95% Confidence Intervals)....21
Supplemental Table S17. The relative performance changes of the ensemble model after ablating the CNN base model....22
Supplemental Table S18. The relative performance changes of the ensemble model after ablating the Bi-LSTM base model....22
Supplemental Table S19. The relative performance changes of the ensemble model after ablating the Transformer base model....22
Supplemental Table S20. Metrics for EnDeep4mC, Hyb4mC, and EpiTEAmDNA across all six species....23
Supplemental Table S21. ONT data statistics of the three test species datasets....24
Supplemental Table S22. Performance metrics of EnDeep4mC on the datasets of three test species....24
Supplemental Methods....25

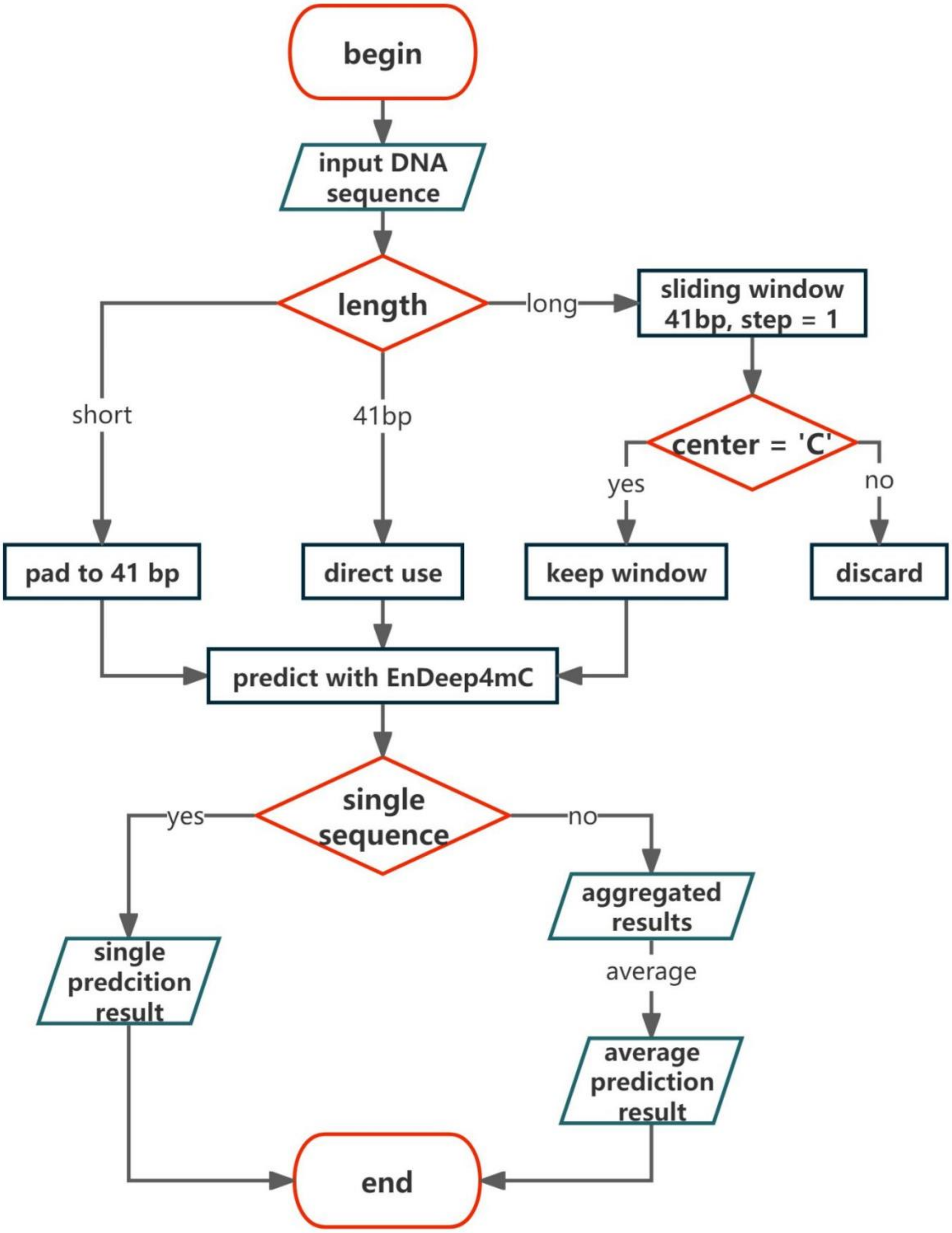

Supplemental Figure S1. Processing Pipeline for Variable-Length Sequences.

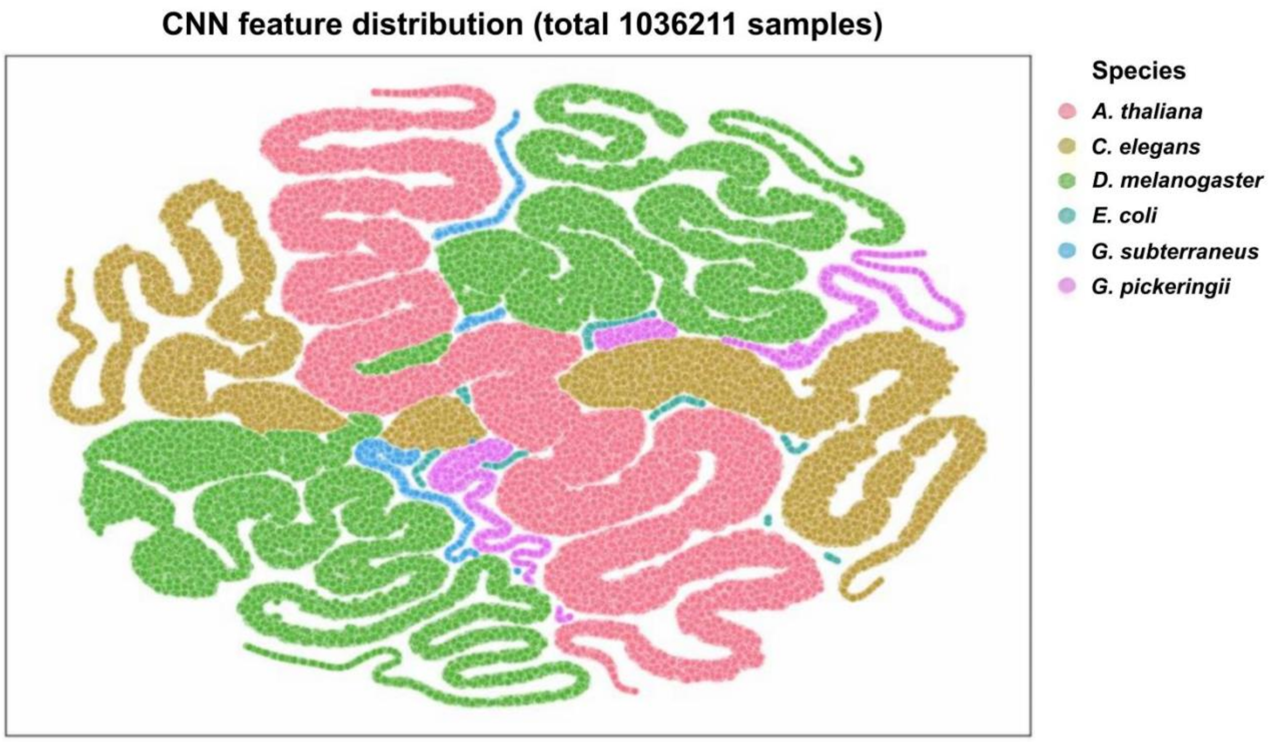

Supplemental Figure S2. The t-SNE visualization of feature distributions of six species after data processing. (Take CNN as an example.)

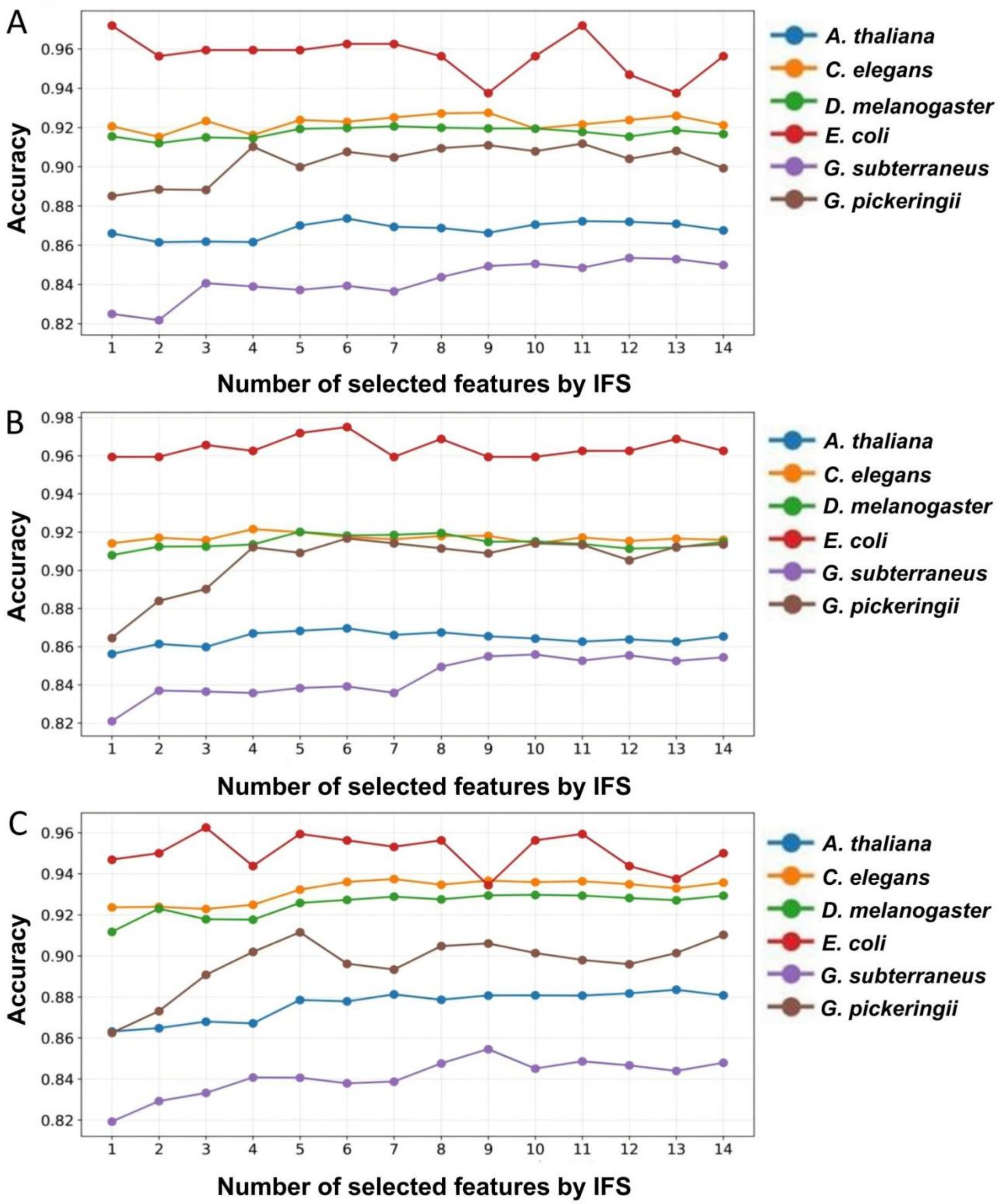

Supplemental Figure S3. The distribution of the best feature sets of three base models on benchmark datasets of six species. (A) CNN model. (B) Bi-LSTM model. (C) Transformer model.

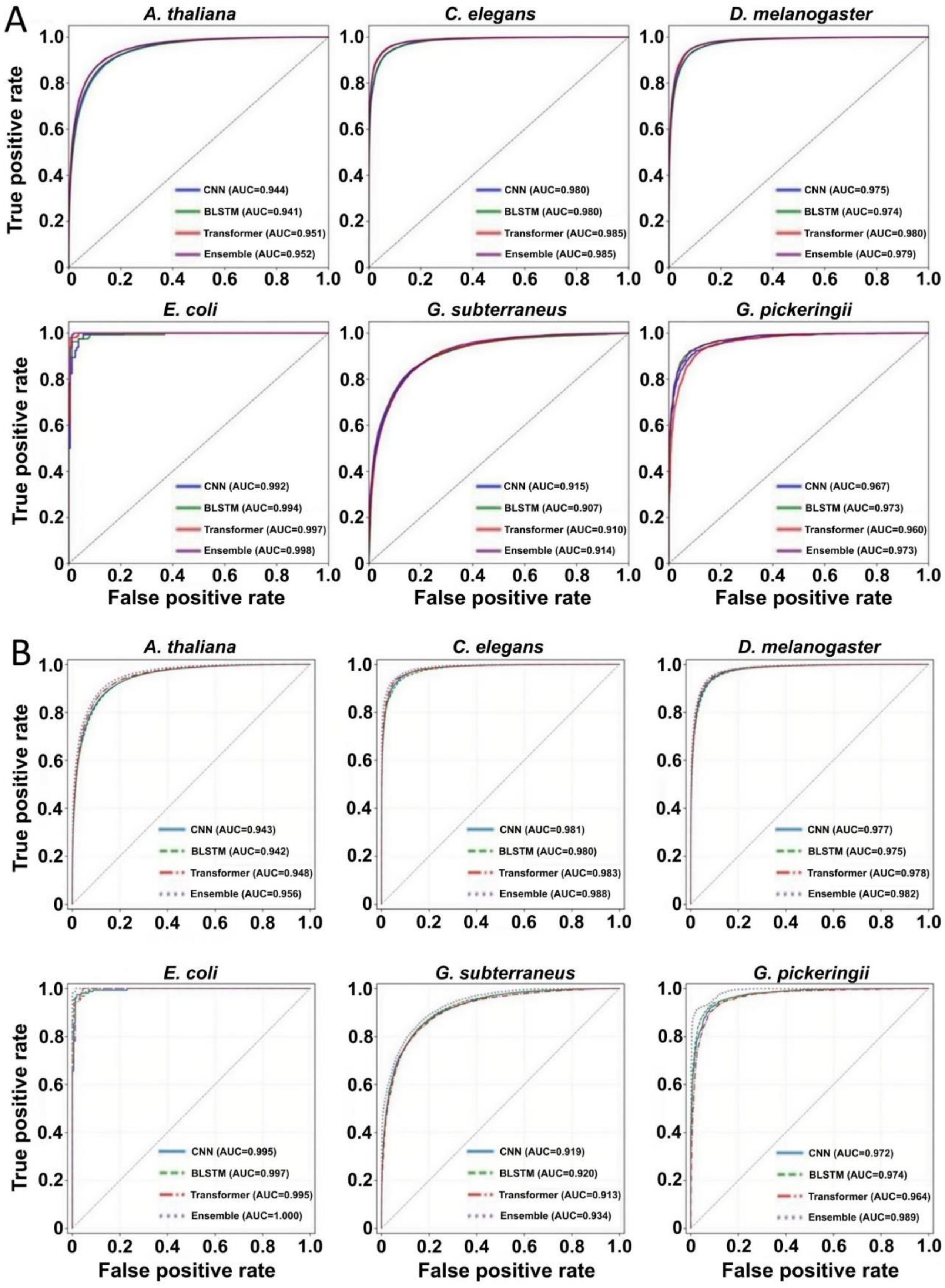

Supplemental Figure S4. ROC curves of training the deep models on the benchmark datasets of six species. (A) Independent test set validation. (B) 5-fold cross-validation.

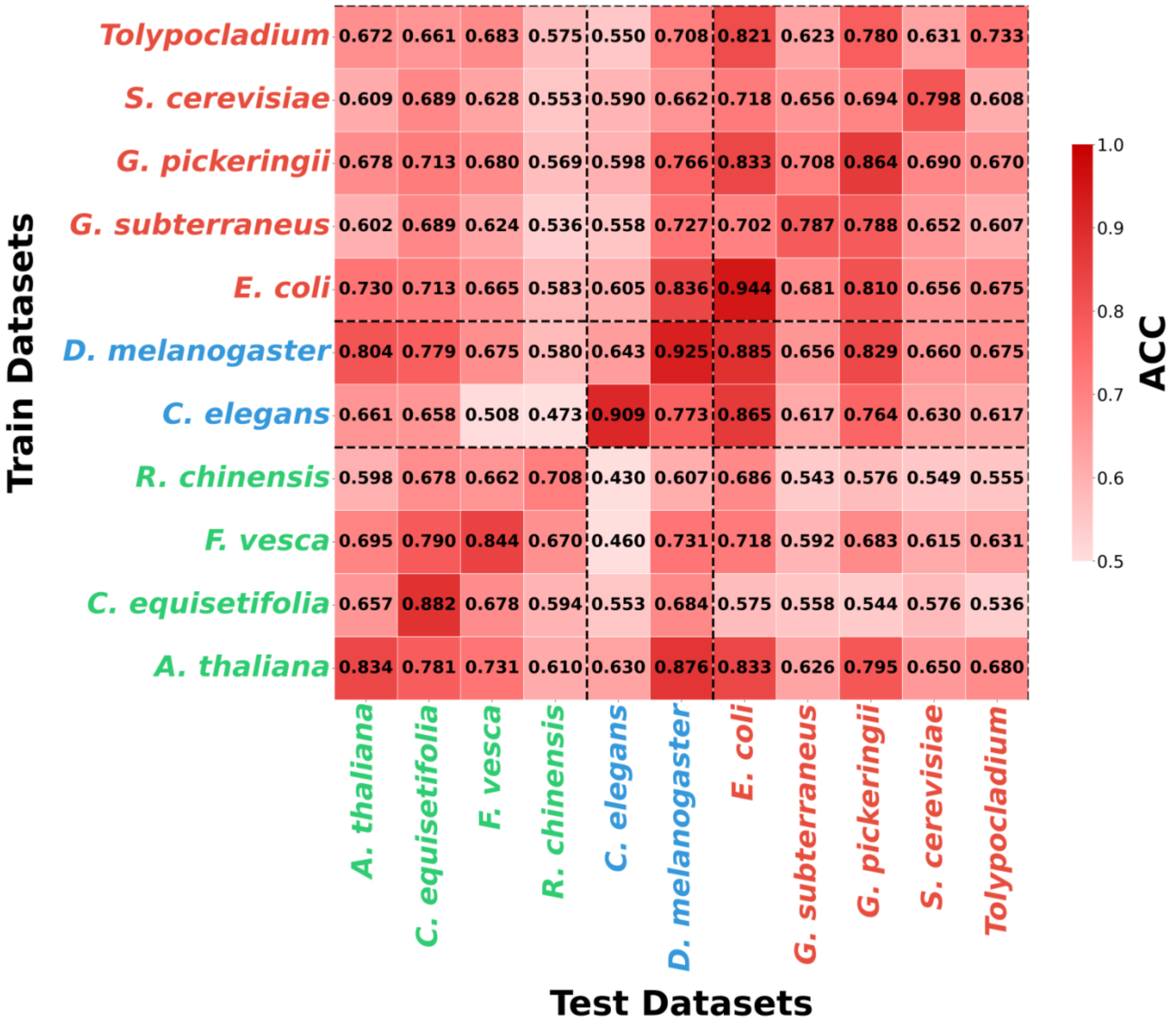

Supplemental Figure S5. Cross-prediction on datasets of 10 species using ACC as indicator. There are some datasets with the same name as the original benchmark datasets, but different from which in data, so a “2” is added after their name to distinguish them.

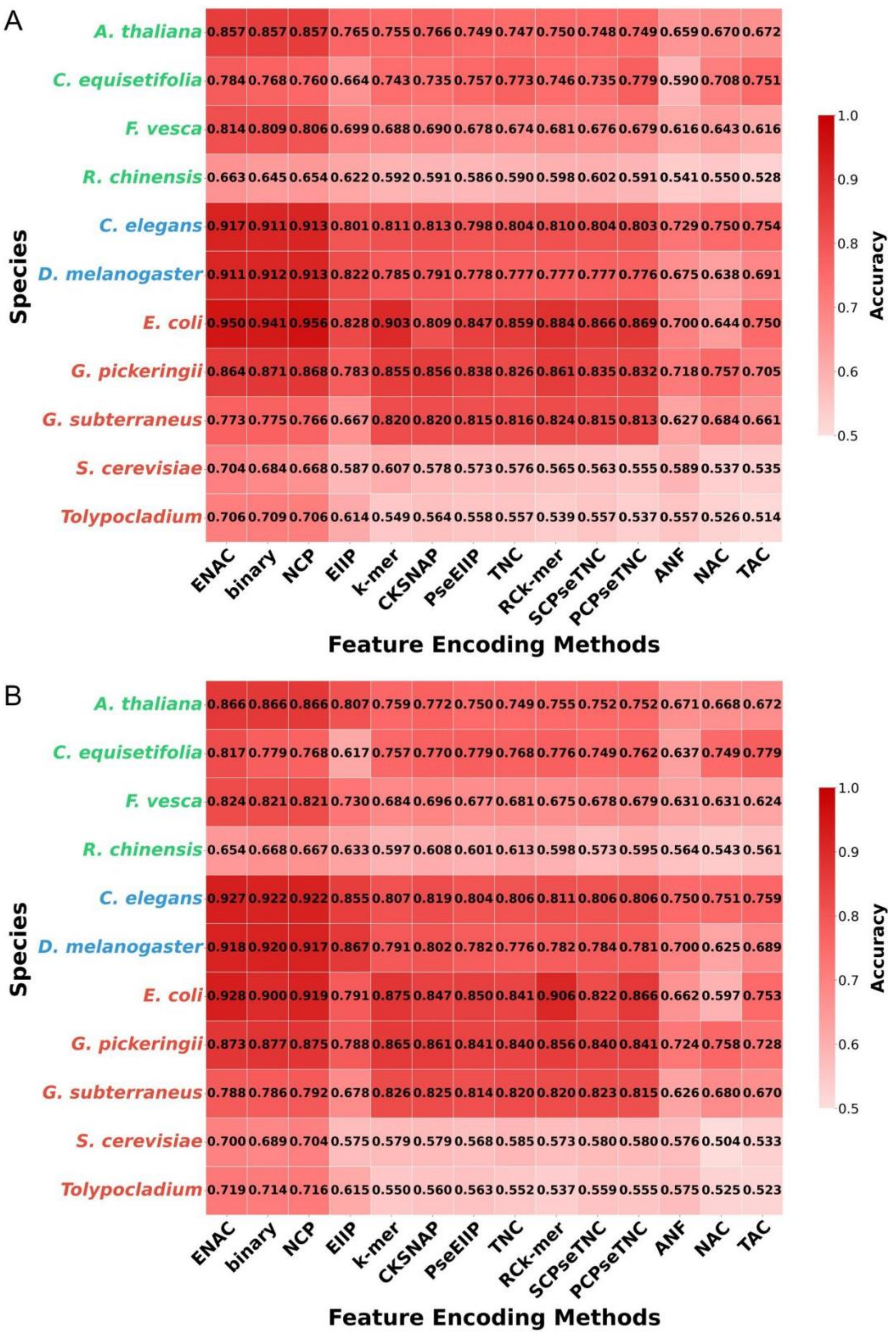

Supplemental Figure S6. Prediction performance on independent test sets of all 16 species after encoding by 14 feature encoding methods respectively. (A) Using Bi-LSTM model. (B) Using Transformer model.

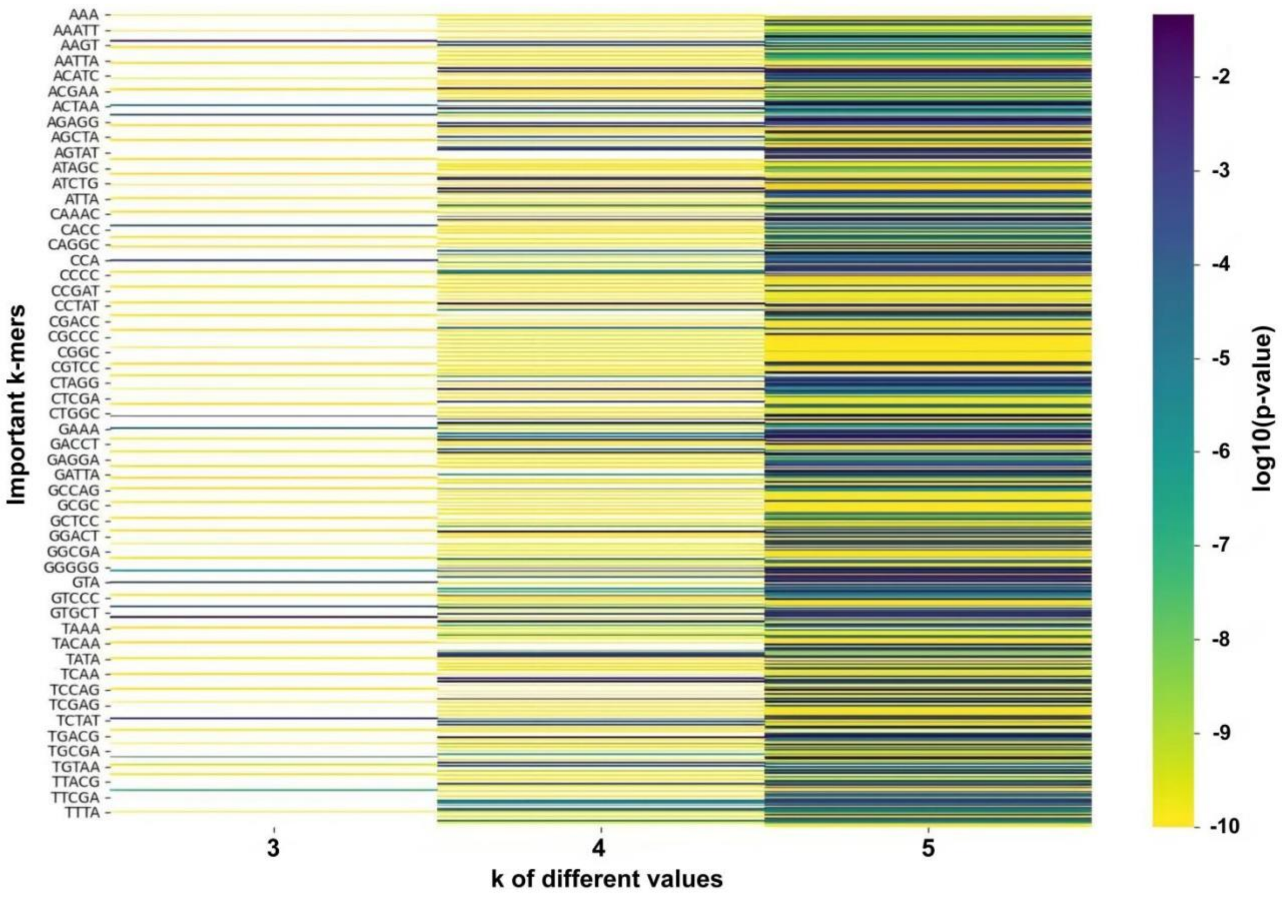

Supplemental Figure S7. The significance level distribution heatmap of k-mer at different k values in the k-mer analysis experiment. The X-axis represents different values of k in the k-mer (k = 3,4,5). The Y-axis represents a reference list of specific k-mer sequences, arranged alphabetically from top to bottom. The p-value of Fisher's exact test indicates the significance of the difference in the distribution of the k-mer between prokaryotes and eukaryotes. The heatmap's color scale is log10-transformed p-values, with smaller values indicating more significant differences (dark yellow). White areas (p-value > 0.1) indicates no significant difference.

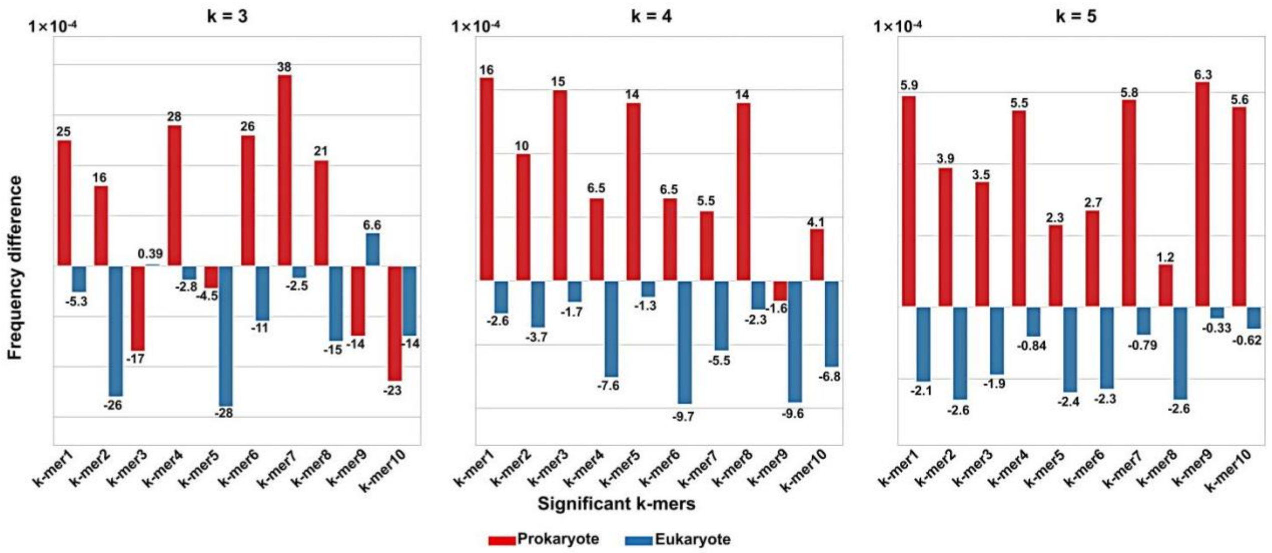

Supplemental Figure S8. Bar charts of the Top 10 significant k-mer frequency differences among eukaryotic/prokaryotic groups in the k-mer analysis experiment. The X-axis represents the top 10 most significant k-mer sequences. The Y-axis represents the frequency difference between eukaryotic and prokaryotic groups, and the value is the positive sample frequency minus the negative sample frequency of the k-mer in different groups.

Supplemental Table S1. Data Statistics of the Benchmark Datasets for 4mC Site Prediction Across Multiple Species. Datasets with ‘2’ are held-out subsets from the six benchmark species to assess zero-shot transfer to previously unseen data.

Datasets	*Train_Pos*	*Train_Neg*	*Test_Pos*	*Test_Neg*
A.thaliana	74662	74662	50966	50966
A.thaliana2	63720	63720	11307	11307
C.elegans	56770	56770	12147	12147
C.elegans2	55729	55729	2667	2667
C.equisetifolia	183	183	183	183
D.melanogaster	81289	81289	28000	28000
D.melanogaster2	53970	53970	3684	3684
E.coli	1908	1908	160	160
E.coli2	1941	1941	126	126
F.vesca	7899	7899	7898	7898
G.pickeringii	3761	3761	1926	1926
G.pickeringii2	4514	4514	1210	1210
G.subterraneus	7064	7064	7813	7813
G.subterraneus2	10584	10780	5263	5263
R.chinensis	1937	1937	483	483
S.cerevisiae	990	990	989	989
Tolypocladium	7664	7664	7663	7663

Supplemental Table S2. Diversity analysis for base models

Metrics	model pair	*A.thaliana*	*C.elegans*	*D.melanogaster*	*E.coli*	*G.subterraneus*	*G.pickeringii*
K Values	CNN_BLSTM	-0.3309	-0.1850	-0.0334	-0.2403	-5.6044	-5.6875
	CNN_Transformer	-0.0160	-0.1273	-0.1634	-0.2308	-2.9964	-4.8899
	BLSTM_Transformer	-2.8000	-4.1383	-5.2964	-2.4043	-3.1078	-6.0549
Correlation	CNN_BLSTM	-0.2722	-0.5932	-0.5743	-0.1848	-0.0041	0.0928
	CNN_Transformer	-0.1466	-0.5567	-0.6234	-0.2036	0.0075	0.0456
	BLSTM_Transformer	0.1966	0.0492	0.0853	0.3796	0.0215	-0.0192
Mean Errors	CNN_BLSTM	0.2476	0.2584	0.2691	0.2078	0.2773	0.1646
	CNN_Transformer	0.2768	0.2666	0.2583	0.2031	0.2821	0.1699
	BLSTM_Transformer	0.2109	0.1201	0.1148	0.1578	0.2745	0.1593
Disagreement Measures	CNN_BLSTM	0.6318	0.7930	0.7870	0.5906	0.5011	0.4756
	CNN_Transformer	0.5733	0.7766	0.8086	0.6000	0.4975	0.4873
	BLSTM_Transformer	0.4335	0.4854	0.4769	0.3656	0.4930	0.5049
Q Statistics	CNN_BLSTM	-0.5233	-0.8885	-0.8642	-0.3642	-0.0155	0.3498
	CNN_Transformer	-0.2871	-0.8555	-0.9068	-0.3981	0.0228	0.1634
	BLSTM_Transformer	0.5600	0.1655	0.3131	0.9373	0.0658	-0.0749

Supplemental Table S3. Architecture and Hyperparameters of Base Deep Learning Models

Component	CNN	Bi-LSTM	Transformer
Input	(1, feature_dim)	(1, feature_dim)	(None, feature_dim)
Layer 1	Conv1D(256,1)+BN	BiLSTM(128)	MultiHead(8 heads)
Layer 2	SepConv1D(128,3)+Pool	BN	FFN(512)+LayerNorm
Layer 3	Conv1D(64,1)	BiLSTM(64)	2 encoder layers
Pooling	GlobalMaxPool	-	GlobalAvgPool
Dense	Dense(128)	Dense(64)	Dense(128)
Output	Dense(1, sigmoid)	Dense(1, sigmoid)	Dense(1, sigmoid)
Regularization	L2(0.001), Drop(0.3)	L2(0.001), Drop(0.2), RecDrop(0.1)	L2(0.001), Drop(0.1)
Optimizer	Adam(lr=0.001, clip=1.0)	Adam(lr=0.001, clip=1.0)	Adam(lr=0.001)

Supplemental Table S4. Configuration of Ensemble Learning Framework

Component	XGBoost Configuration	LightGBM Configuration	Meta-Learner Configuration
Model Type	XGBClassifier	LGBMClassifier	LogisticRegression
Number of Trees	n_estimators=500	n_estimators=300	-
Learning Rate	0.05	0.05	-
Depth	max_depth=7	num_leaves=63	-
Regularization	gamma=0.1, subsample=0.8	reg_alpha=0.2, reg_lambda=0.2	C=0.6, l1_ratio=0.5
Others	colsample_bytree=0.8	min_child_samples=20	penalty='elasticnet', solver='saga'

Supplemental Table S5. Training Configuration Parameters

Parameter Category	Specific Configuration
Cross-Validation	StratifiedKFold(n_splits=5, shuffle=True, random_state=42)
Early Stopping	EarlyStopping(monitor='val_accuracy', patience=15, restore_best_weights=True)
Data Augmentation	SMOTE oversampling + Gaussian noise(σ=0.05)
Standardization	StandardScaler()
Class Weights	compute_class_weight('balanced')
Callbacks	ModelCheckpoint + Custom TrainingMonitor

Supplemental Table S6. Summary of the candidate feature encoding schemes in this paper

Feature Encoding Schemes	*Encoding Type*	*Full Name*
ENAC	Nucleic Acid Composition	Enhanced Nucleic Acid Composition
k-mer	Nucleic Acid Composition	k-mer Frequency
NAC	Composition	Nucleotide Composition
TNC	Composition	Trinucleotide Composition
Binary	Binary	Binary Encoding
NCP	physicochemical Properties	Nucleotide Conformation Parameters
EIIP	physicochemical Properties	Electron-Ion Interaction Potential
PseEIIP	physicochemical Properties	Pseudo Electron-Ion Interaction Potential
ANF	Location Information	Accumulated Nucleotide Frequency
SCPseTNC	Pseudo Nucleotide Composition	Serial Correlation Pseudo Trinucleotide Composition
PCPseTNC	Pseudo Nucleotide Composition	Physicochemical Correlation Pseudo Trinucleotide Composition
TAC	Autocorrelated	Trinucleotide Autocorrelation
RCk-mer	Autocorrelated	Reverse Complement K-mer
CKSNAP	Autocorrelated	Combined K-mer and Nucleotide Adjacency Profile

Supplemental Table S7. Feature sets of the benchmark datasets of 6 species selected by the dynamic feature selection (DFS) framework

Model	*Species*	*Best_N(n*)*	*Optimal_Features*
CNN	*A.thaliana*	6	NCP, ENAC, binary, EIIP, CKSNAP, k-mer
CNN	*C.elegans*	9	ENAC, binary, NCP, EIIP, k-mer, CKSNAP, TNC, SCPseTNC, PCPseTNC
CNN	*D.melanogaster*	7	NCP, binary, ENAC, EIIP, CKSNAP, k-mer, TNC
CNN	*E.coli*	1	ENAC
CNN	*G.subterraneus*	12	CKSNAP, SCPseTNC, k-mer, PCPseTNC, PseEIIP, TNC, RCk-mer, ENAC, NCP, binary, NAC, EIIP
CNN	*G.pickeringii*	11	binary, ENAC, NCP, CKSNAP, k-mer, RCk-mer, SCPseTNC, PCPseTNC, TNC, PseEIIP, EIIP
BLSTM	*A.thaliana*	6	ENAC, binary, NCP, CKSNAP, EIIP, k-mer
BLSTM	*C.elegans*	4	ENAC, binary, NCP, CKSNAP
BLSTM	*D.melanogaster*	5	binary, ENAC, NCP, EIIP, CKSNAP
BLSTM	*E.coli*	6	ENAC, NCP, binary, RCk-mer, k-mer, SCPseTNC
BLSTM	*G.subterraneus*	10	CKSNAP, k-mer, RCk-mer, TNC, PCPseTNC, PseEIIP, SCPseTNC, binary, NCP, ENAC
BLSTM	*G.pickeringii*	6	NCP, binary, ENAC, CKSNAP, RCk-mer, k-mer
Transformer	*A.thaliana*	13	NCP, binary, ENAC, EIIP, CKSNAP, k-mer, SCPseTNC, PCPseTNC, TNC, PseEIIP, RCk-mer, TAC, ANF
Transformer	*C.elegans*	7	ENAC, NCP, binary, EIIP, CKSNAP, k-mer, RCk-mer
Transformer	*D.melanogaster*	10	NCP, ENAC, binary, EIIP, CKSNAP, k-mer, SCPseTNC, TNC, PCPseTNC, PseEIIP
Transformer	*E.coli*	3	ENAC, binary, NCP
Transformer	*G.subterraneus*	9	PseEIIP, CKSNAP, k-mer, SCPseTNC, PCPseTNC, TNC, RCk-mer, ENAC, binary
Transformer	*G.pickeringii*	5	NCP, binary, ENAC, CKSNAP, k-mer

Supplemental Table S8. The relative accuracy changes of the CNN model after ablating each feature selected by the DFS framework (in descending order of importance)

CNN	*A.thaliana*	*C.elegans*	*D.melanogaster*	*E.coli*	*G.subterraneus*	*G.pickeringii*
NCP	-0.0259	-0.0120	-0.0134	-	-0.0220	-0.0091
ENAC	-0.0252	-0.0089	-0.0136	-	-0.0170	-0.0039
binary	-0.0270	-0.0124	-0.0125	-	-0.0172	-0.0026
EIIP	-0.0253	-0.0067	-0.0124	-	-0.0156	-0.0055
CKSNAP	-0.0255	-0.0103	-0.0126	-	-0.0282	-0.0145
k-mer	-0.0299	-0.0103	-0.0091	-	-0.0117	-0.0083
TNC	-	-0.0083	-0.0117	-	-0.0191	-0.0055
SCPseTNC	-	-0.0068	-	-	-0.0166	-0.0039
PCPseTNC	-	-0.0080	-	-	-0.0141	-0.0016
PseEIIP	-	-	-	-	-0.0182	-0.0086
RCk-mer	-	-	-	-	-0.0124	-0.0039
NAC	-	-	-	-	-0.0122	-

Supplemental Table S9. The relative accuracy changes of the Bi-LSTM model after ablating each feature selected by the DFS framework (in descending order of importance)

Bi-LSTM	*A.thaliana*	*C.elegans*	*D.melanogaster*	*E.coli*	*G.subterraneus*	*G.pickeringii*
ENAC	-0.0205	-0.0130	-0.0154	-	-0.0012	0.0021
binary	-0.0212	-0.0099	-0.0134	-0.0125	0.0003	0.0080
NCP	-0.0202	-0.0081	-0.0114	-0.0094	0.0003	0.0044
CKSNAP	-0.0213	-0.0160	-0.0198	-	-0.0019	0.0013
EIIP	-0.0198	-	-0.0153	-	-	-
k-mer	-0.0254	-	-	0.0031	0.0021	0.0062
RCk-mer	-	-	-	0.0031	0.0002	0.0036
SCPseTNC	-	-	-	0.0062	0.0042	-
TNC	-	-	-	-	0.0005	-
PCPseTNC	-	-	-	-	0.0006	-
PseEIIP	-	-	-	-	0.0014	-

Supplemental Table S10. The relative accuracy changes of the Transformer model after ablating each feature selected by the DFS framework (in descending order of importance)

Transformer	*A.thaliana*	*C.elegans*	*D.melanogaster*	*E.coli*	*G.subterraneus*	*G.pickeringii*
NCP	-0.0030	-0.0042	-0.0008	-	-	0.0005
binary	-0.0043	-0.0018	-0.0004	-	-0.0123	0.0021
ENAC	-0.0051	-0.0029	-0.0005	-0.0063	-0.0145	0.0018
EIIP	-0.0050	-0.0023	0.0004	-	-	-
CKSNAP	-0.0054	-0.0039	-0.0009	-	-0.0148	-0.0049
k-mer	-0.0049	-0.0038	0.0010	-	-0.0074	-0.0005
SCPseTNC	-0.0046	-	-0.0002	-	-0.0054	-
PCPseTNC	-0.0052	-	-0.0005	-	-0.0088	-
TNC	-0.0051	-	0.0002	-	-0.0099	-
PseEIIP	-0.0059	-	0.0003	-	-0.0063	-

RCk-mer	-0.0040	-0.0028	-	-	-0.0117	-
TAC	-0.0042	-	-	-	-	-
ANF	-0.0036	-	-	-	-	-

Supplemental Table S11. Statistical performance analysis of models on *A. thaliana* (with 95% Confidence Intervals)

A. thaliana	Metric	Mean ± Std	95% Confidence Interval
CNN	ACC	0.8727 ± 0.0009	0.8717-0.8738
	SN	0.8933 ± 0.0103	0.8805-0.9061
	SP	0.8522 ± 0.0113	0.8382-0.8662
	MCC	0.7489	-
	AUC	0.9453 ± 0.0005	0.9446-0.9459
	F1 Score	0.8758	-
Bi-LSTM	ACC	0.8702 ± 0.0008	0.8691-0.8712
	SN	0.8781 ± 0.0039	0.8733-0.8829
	SP	0.8623 ± 0.0028	0.8587-0.8658
	MCC	0.7423	-
	AUC	0.9423 ± 0.0002	0.9419-0.9426
	F1 Score	0.8727	-
Transformer	ACC	0.8789 ± 0.0009	0.8778-0.8801
	SN	0.8962 ± 0.0041	0.8911-0.9012
	SP	0.8617 ± 0.0054	0.8551-0.8684
	MCC	0.7587	-
	AUC	0.9491 ± 0.0003	0.9487-0.9495
	F1 Score	0.8811	-
EnDeep4mC	ACC	0.9133 ± 0.0003	0.9130-0.9136
	SN	0.9205 ± 0.0031	0.9167-0.9243
	SP	0.9061 ± 0.0034	0.9019-0.9103
	MCC	0.8267 ± 0.0006	0.8260-0.8274
	AUC	0.9697 ± 0.0003	0.9693-0.9701
	F1 Score	0.9133 ± 0.0004	0.9128-0.9138

Supplemental Table S12. Statistical performance analysis of models on *C. elegans* (with 95% Confidence Intervals)

C. elegans	Metric	Mean ± Std	95% Confidence Interval
CNN	ACC	0.9252 ± 0.0027	0.9218-0.9286
	SN	0.9559 ± 0.0043	0.9505-0.9612
	SP	0.8946 ± 0.0083	0.8842-0.9049
	MCC	0.8529	-
	AUC	0.9812 ± 0.0004	0.9807-0.9816
	F1 Score	0.9278	-
Bi-LSTM	ACC	0.9231 ± 0.0014	0.9214-0.9249
	SN	0.9514 ± 0.0018	0.9492-0.9537
	SP	0.8948 ± 0.0032	0.8908-0.8989
	MCC	0.8461	-
	AUC	0.9792 ± 0.0002	0.9789-0.9795
	F1 Score	0.9246	-
Transformer	ACC	0.9296 ± 0.0015	0.9278-0.9315
	SN	0.9589 ± 0.0027	0.9556-0.9622
	SP	0.9003 ± 0.0045	0.8947-0.9060
	MCC	0.859	-
	AUC	0.9829 ± 0.0003	0.9826-0.9833
	F1 Score	0.9308	-
EnDeep4mC	ACC	0.9571 ± 0.0004	0.9567-0.9575
	SN	0.9594 ± 0.0010	0.9582-0.9606
	SP	0.9548 ± 0.0010	0.9537-0.9559
	MCC	0.9142 ± 0.0008	0.9134-0.9150
	AUC	0.9914 ± 0.0002	0.9912-0.9916
	F1 Score	0.9572 ± 0.0004	0.9567-0.9577

Supplemental Table S13. Statistical performance analysis of models on *D. melanogaster* (with 95% Confidence Intervals)

D. melanogaster	Metric	Mean ± Std	95% Confidence Interval
CNN	ACC	0.9191 ± 0.0008	0.9181-0.9201
	SN	0.9530 ± 0.0039	0.9482-0.9579
	SP	0.8851 ± 0.0052	0.8787-0.8916
	MCC	0.8401	-
	AUC	0.9758 ± 0.0002	0.9755-0.9761
	F1 Score	0.9216	-
Bi-LSTM	ACC	0.9169 ± 0.0015	0.9150-0.9187
	SN	0.9502 ± 0.0035	0.9459-0.9545
	SP	0.8836 ± 0.0062	0.8759-0.8913
	MCC	0.8379	-
	AUC	0.9738 ± 0.0001	0.9737-0.9740
	F1 Score	0.9205	-
Transformer	ACC	0.9246 ± 0.0023	0.9217-0.9274
	SN	0.9540 ± 0.0036	0.9495-0.9585
	SP	0.8951 ± 0.0081	0.8851-0.9052
	MCC	0.8512	-
	AUC	0.9778 ± 0.0002	0.9775-0.9781
	F1 Score	0.927	-
EnDeep4mC	ACC	0.9412 ± 0.0005	0.9406-0.9418
	SN	0.9508 ± 0.0018	0.9487-0.9529
	SP	0.9316 ± 0.0015	0.9299-0.9333
	MCC	0.8826 ± 0.0009	0.8815-0.8837
	AUC	0.9842 ± 0.0002	0.9840-0.9844
	F1 Score	0.9418 ± 0.0005	0.9412-0.9424

Supplemental Table S14. Statistical performance analysis of models on *E. coli* (with 95% Confidence Intervals)

E. coli	Metric	Mean ± Std	95% Confidence Interval
CNN	ACC	0.9619 ± 0.0114	0.9478-0.9760
	SN	0.9513 ± 0.0252	0.9200-0.9825
	SP	0.9725 ± 0.0056	0.9656-0.9794
	MCC	0.9438	-
	AUC	0.9933 ± 0.0011	0.9919-0.9946
	F1 Score	0.9718	-
Bi-LSTM	ACC	0.9644 ± 0.0078	0.9546-0.9741
	SN	0.9437 ± 0.0077	0.9342-0.9533
	SP	0.9850 ± 0.0105	0.9720-0.9980
	MCC	0.9251	-
	AUC	0.9972 ± 0.0003	0.9968-0.9976
	F1 Score	0.9623	-
Transformer	ACC	0.9675 ± 0.0098	0.9554-0.9796
	SN	0.9613 ± 0.0068	0.9527-0.9698
	SP	0.9737 ± 0.0135	0.9569-0.9906
	MCC	0.9376	-
	AUC	0.9947 ± 0.0004	0.9941-0.9952
	F1 Score	0.9686	-
EnDeep4mC	ACC	0.9973 ± 0.0017	0.9951-0.9995
	SN	0.9969 ± 0.0018	0.9947-0.9991
	SP	0.9976 ± 0.0017	0.9955-0.9997
	MCC	0.9945 ± 0.0034	0.9903-0.9987
	AUC	0.9999 ± 0.0000	0.9999-0.9999
	F1 Score	0.9973 ± 0.0017	0.9951-0.9995

Supplemental Table S15. Statistical performance analysis of models on *G. subterraneus* (with 95% Confidence Intervals)

G. subterraneus	Metric	Mean ± Std	95% Confidence Interval
CNN	ACC	0.8525 ± 0.0038	0.8478-0.8572
	SN	0.8725 ± 0.0163	0.8523-0.8927
	SP	0.8325 ± 0.0236	0.8033-0.8618
	MCC	0.7102	-
	AUC	0.9334 ± 0.0009	0.9323-0.9346
	F1 Score	0.852	-
Bi-LSTM	ACC	0.8551 ± 0.0017	0.8530-0.8572
	SN	0.8545 ± 0.0035	0.8502-0.8589
	SP	0.8557 ± 0.0032	0.8518-0.8596
	MCC	0.7142	-
	AUC	0.9309 ± 0.0014	0.9291-0.9327
	F1 Score	0.8578	-
Transformer	ACC	0.8514 ± 0.0046	0.8457-0.8570
	SN	0.8527 ± 0.0182	0.8301-0.8753
	SP	0.8500 ± 0.0111	0.8362-0.8638
	MCC	0.7024	-
	AUC	0.9282 ± 0.0018	0.9260-0.9305
	F1 Score	0.8513	-
EnDeep4mC	ACC	0.9349 ± 0.0032	0.9305-0.9393
	SN	0.9323 ± 0.0025	0.9292-0.9354
	SP	0.9375 ± 0.0040	0.9324-0.9426
	MCC	0.8698 ± 0.0066	0.8617-0.8779
	AUC	0.9786 ± 0.0014	0.9768-0.9804
	F1 Score	0.9347 ± 0.0033	0.9303-0.9391

Supplemental Table S16. Statistical performance analysis of models on *G. pickeringii* (with 95% Confidence Intervals)

G. pickeringii	Metric	Mean ± Std	95% Confidence Interval
CNN	ACC	0.9075 ± 0.0031	0.9036-0.9114
	SN	0.9437 ± 0.0109	0.9302-0.9573
	SP	0.8712 ± 0.0134	0.8546-0.8878
	MCC	0.8233	-
	AUC	0.9722 ± 0.0006	0.9714-0.9729
	F1 Score	0.9138	-
Bi-LSTM	ACC	0.9180 ± 0.0022	0.9153-0.9207
	SN	0.9384 ± 0.0037	0.9338-0.9430
	SP	0.8976 ± 0.0021	0.8951-0.9002
	MCC	0.8345	-
	AUC	0.9742 ± 0.0003	0.9739-0.9746
	F1 Score	0.9186	-
Transformer	ACC	0.9107 ± 0.0032	0.9067-0.9147
	SN	0.9346 ± 0.0071	0.9258-0.9433
	SP	0.8868 ± 0.0115	0.8725-0.9011
	MCC	0.8241	-
	AUC	0.9709 ± 0.0014	0.9691-0.9726
	F1 Score	0.9127	-
EnDeep4mC	ACC	0.9729 ± 0.0030	0.9691-0.9767
	SN	0.9740 ± 0.0038	0.9693-0.9787
	SP	0.9718 ± 0.0023	0.9690-0.9746
	MCC	0.9457 ± 0.0060	0.9385-0.9529
	AUC	0.9937 ± 0.0006	0.9930-0.9944
	F1 Score	0.9729 ± 0.0030	0.9691-0.9767

Supplemental Table S17. The relative performance changes of the ensemble model after ablating the CNN base model

CNN	ACC	SN	SP	F1	MCC	AUC
A.thaliana	0.0002	0.0005	-0.0002	0.0002	0.0003	0
C.elegans	-0.0011	0	-0.0022	-0.0011	-0.0023	-0.0002
D.melanogaster	-0.0003	-0.0004	-0.0003	-0.0003	-0.0007	-0.0002
E.coli	-0.0032	-0.0025	-0.0038	-0.0032	-0.0065	-0.0004
G.subterraneus	-0.0077	-0.0028	-0.0126	-0.0072	-0.0172	-0.0047
G.pickeringii	0.0014	0.0005	0.0022	0.0013	0.0028	0.0005

Supplemental Table S18. The relative performance changes of the ensemble model after ablating the Bi-LSTM base model

Bi-LSTM	ACC	SN	SP	F1	MCC	AUC
A.thaliana	-0.0118	-0.0121	-0.0115	-0.0118	-0.026	-0.0057
C.elegans	-0.0052	-0.0039	-0.0064	-0.0051	-0.0108	-0.0011
D.melanogaster	-0.0076	-0.0067	-0.0086	-0.0075	-0.0161	-0.0027
E.coli	-0.0019	0	-0.0038	-0.0019	-0.0038	-0.0012
G.subterraneus	-0.0047	-0.0033	-0.006	-0.0045	-0.0104	-0.0046
G.pickeringii	-0.0175	-0.0218	-0.0132	-0.0177	-0.0364	-0.0049

Supplemental Table S19. The relative performance changes of the ensemble model after ablating the Transformer base model

Transformer	ACC	SN	SP	F1	MCC	AUC
A.thaliana	-0.0016	-0.0019	-0.0012	-0.0016	-0.0034	-0.0018
C.elegans	-0.0041	-0.0042	-0.004	-0.0041	-0.0086	-0.0015
D.melanogaster	-0.0022	-0.001	-0.0034	-0.0021	-0.0046	-0.0013
E.coli	-0.0032	-0.0038	-0.0026	-0.0032	-0.0065	0.0001
G.subterraneus	-0.0019	-0.0015	-0.0023	-0.0019	-0.0042	-0.0015
G.pickeringii	0.0027	0.0011	0.0044	0.0027	0.0057	-0.0005

Supplemental Table S20. Metrics for EnDeep4mC, Hyb4mC, and EpiTEAmDNA across all six species

Dataset	Algorithm	ACC	SN	SP	MCC	AUC	F1 Score
C. elegans	EpiTEAmDNA	0.9432	**0.9687**	0.9217	0.8876	0.9883	0.9446
	Hyb4mC(5-CV)	0.9340	0.9500	0.9081	0.8464	0.9850	0.9360
	EnDeep4mC	**0.9571**	0.9594	**0.9548**	**0.9142**	**0.9914**	**0.9572**
D. melanogaster	EpiTEAmDNA	0.9358	**0.9644**	0.9123	0.8731	0.9832	0.9376
	Hyb4mC(5-CV)	0.9260	0.9451	0.8930	0.8394	0.9790	0.9281
	EnDeep4mC	**0.9412**	0.9508	**0.9316**	**0.8826**	**0.9842**	**0.9418**
A. thaliana	EpiTEAmDNA	0.8829	0.8950	0.8738	0.7660	0.9513	0.8843
	Hyb4mC(5-CV)	0.8730	0.8951	0.8510	0.7115	0.9460	0.8761
	EnDeep4mC	**0.9133**	**0.9205**	**0.9061**	**0.8267**	**0.9697**	**0.9139**
E. coli	EpiTEAmDNA	0.9875	0.9875	0.9875	0.9875	0.9995	0.9750
	Hyb4mC(5-CV)	0.9756	0.9513	**1.0000**	0.9524	**1.0000**	0.9750
	EnDeep4mC	**0.9973**	**0.9969**	0.9976	**0.9945**	0.9999	**0.9973**
G. subterraneus	EpiTEAmDNA	0.8642	0.8472	0.8770	0.7288	0.9398	0.8618
	Hyb4mC(5-CV)	0.8156	0.7914	0.8398	0.6321	0.8803	0.8111
	EnDeep4mC	**0.9349**	**0.9323**	**0.9375**	**0.8698**	**0.9786**	**0.9347**
G. pickeringii	EpiTEAmDNA	0.9338	0.9486	0.9213	0.8680	0.9826	0.9348
	Hyb4mC(5-CV)	0.8900	0.9215	0.8583	0.7816	0.9589	0.8937
	EnDeep4mC	**0.9729**	**0.9740**	**0.9717**	**0.9457**	**0.9937**	**0.9729**

Supplemental Table S21. ONT data statistics of the three test species datasets

Datasets	Train_Pos	Train_Neg	Test_Pos	Test_Neg
E. faecium	2284	2284	1524	1524
K. pneumoniae	10884	10884	7257	7257
L.monocytogenes	6385	6385	4257	4257

Supplemental Table S22. Performance metrics of EnDeep4mC on the datasets of three test species

CNN	ACC	SN	SP	F1-Score	MCC	AUC
E. faecium	0.994423	0.990814	0.998031	0.994402	0.988871	0.999068
K. pneumoniae	0.998829	0.998622	0.999035	0.998828	0.997658	0.999964
L. monocytogenes	0.993070	0.992248	0.993892	0.993065	0.986142	0.999387
BLSTM	ACC	SN	SP	F1-Score	MCC	AUC
E. faecium	0.994006	0.989390	0.998622	0.993978	0.988054	0.999430
K. pneumoniae	0.994006	0.989390	0.998622	0.993978	0.988054	0.999430
L. monocytogenes	0.977684	0.978858	0.976509	0.977710	0.955370	0.998134
Transformer	ACC	SN	SP	F1-Score	MCC	AUC
E. faecium	0.979659	0.959318	**1.000000**	0.979236	0.960112	0.998424
K. pneumoniae	0.999656	0.999311	**1.000000**	0.999655	0.999311	0.999999
L. monocytogenes	0.999530	0.999060	1.000000	0.999530	0.999061	**0.999997**
Ensemble	ACC	SN	SP	F1-Score	MCC	AUC
E. faecium	**0.999343**	**0.999015**	0.999672	**0.999343**	**0.998687**	**0.999998**
K. pneumoniae	**0.999879**	**0.999862**	0.999897	**0.999879**	**0.999759**	**1**
L. monocytogenes	**0.999883**	**0.999765**	**1**	**0.999882**	**0.999765**	0.999994

Supplemental Methods

Processing Pipeline for Variable-Length Sequences

The EnDeep4mC model is trained on fixed-length sequences (41 bp). To predict 4mC sites in DNA sequences of arbitrary length (e.g., complete bacterial genomes), the following preprocessing pipeline should be applied:

(1) Determine the total length of the input DNA sequence and classify it into one of three categories: shorter than 41 bp, exactly 41 bp, or longer than 41 bp.

(2) Standardize the sequence length accordingly. If the sequence is exactly 41 bp, it can be used directly for prediction. If the sequence is shorter than 41 bp, symmetrically pad both ends with neutral 'N' bases until the total length reaches 41 bp. If the sequence is longer than 41 bp, proceed to sliding window segmentation.

(3) For sequences longer than 41 bp, segment the sequence into overlapping 41-bp fragments using a sliding window with a step size of 1 bp. Retain only those windows where the central position (the 21st base) is a cytosine (C), as windows without a C at the center are biologically irrelevant for 4mC modification prediction.

(4) Input each standardized 41-bp sequence into the EnDeep4mC model to obtain a probability score indicating 4mC modification at the central cytosine.

(5) For predictions on long sequences, the final prediction score for each cytosine position is computed as the average probability of all overlapping windows that cover that position, providing a robust aggregate measure of methylation likelihood across multiple sequence contexts.

ENAC (Enhanced Nucleic Acid Composition)

ENAC is an improvement on the traditional nucleic acid composition encoding (NAC), which considers more characteristic information of nucleic acid sequences and reflects the composition of different bases or base combinations in nucleic acid sequences.

Local window statistics is used to enhance the representation ability of sequence features. For each sliding window of length w, the occurrence frequency of each nucleotide in the window was calculated. For a window $S\ [j: j+w]$ at position j, the frequency of nucleotide α is calculated as follows.

$$f_w(\alpha) = \frac{1}{w} \sum_{i=j}^{j+w-1} \delta\,(s_i, \alpha)$$

Where $i \in [j, j+w-1]$, and δ is the indicator function (take 1 when $S_i = \alpha$, 0 otherwise). Finally, the $4w(L-w+1)$ dimension feature (which L is the sequence length) is generated. In our experiment, we take $w = 2$. The 4D frequencies of A/C/G/T are generated in each window, and the local composition patterns are captured by sliding over the whole sequence.

Binary (Binary Encoding)

Binary Encoding maps each nucleotide to a 4-dimensional one-hot vector representation:

A → [1,0,0,0], C → [0,1,0,0], G → [0,0,1,0], T/U → [0,0,0,1]

This encoding method is simple and direct, and can transform the sequence information into a digital form that is easy to be processed by computers.

NCP (Nucleotide Chemical Property)

NCP is an encoding that converts the physical and chemical parameters related to nucleotide conformation into feature vectors to describe nucleic acid sequences.

Encoding is based on three chemical properties of nucleotides:

Loop structure: purine (A/G) = 1, pyrimidine (C/T) = 0

Hydrogen bond number: strong (C/G) = 1, weak (A/T) = 0

Chemical function: amino (A/C) = 1, ketone (G/T) = 0

Specific assignments are as follows:

A: [1,1,1] (purine, double hydrogen bond, amino group)

C: [0,1,0] (pyrimidine, triple hydrogen bond, amino group)

G: [1,0,0] (purine, trihydrogen bond, ketone group)

T: [0,0,1] (pyrimidine, double hydrogen bond, ketone group)

EIIP (Electron-Ion Interaction Potential)

EIIP is an encoding method that assigns a value to each base based on the electron-ion interaction potential of a nucleotide.

Based on the electron-ion potential value of a nucleotide:

A: 0.1260, C: 0.1340, G: 0.0806, T/U: 0.1335

To map a sequence to a sequence of numbers:

$$EIIP(S) = [v(s_1), v(s_2), \cdots, v(s_L)]$$

Where $v(s_i)$ is the EIIP value of the corresponding nucleotide.

EIIP reflects the physical energy characteristics of DNA through electronic structure features.

k-mer (k-mer Frequency)

k-mer is an encoding method that counts the occurrence frequency of all subsequences of length k in a nucleic acid sequence, and uses it as a feature to describe the encoding mode of the sequence.

In this experiment,we take $k = 4$ for the length of the subsequence, and the normalized frequency of all 4-mer combinations is counted:

$$f(kmer) = \frac{N(kmer)}{L - k + 1} \quad (k = 4)$$

Where $N(kmer)$ is the number of occurrences of k-mers in the sequence. 4^4 = 256-dimensional features are generated to capture local sequential patterns of length 4. For example, the sequence “ACGT” contains 1 “ACGT” k-mer whose frequency is $1/(L-3)$.

CKSNAP (Composition of k-spaced Nucleic Acid Pairs)

CKSNAP is an encoding method based on k-mer and incorporating sequence adjacency information.

In this experiment, the maximum number of gaps $g = 8$, and the frequency of nucleotide pairs separated by 0 to 8 bases is counted:

$$f(XY|g) = \frac{N(\underbrace{X \cdots Y}_{g})}{L - g - 1} \quad (g \epsilon \{0,1,\cdots,8\})$$

Where $X/Y \in \{A, C, G, T\}$, “$X[\ldots g]Y$” means X and Y are separated by g bases. 16×(g+1) = 144-dimensional features (16 nucleotide pairs×9 gaps) were generated. For example, when $g = 2$, “$\mathrm{A} ** \mathrm{C}$” is treated as the interval 2 pairing of A and C.

PseEIIP (Pseudo Electron-Ion Interaction Potential)

PseEIIP is an improved encoding method based on EIIP, which combines trinucleotide frequency and EIIP energy to calculate:

Firstly, the EIIP value of trinucleotide tri is calculated:

$$EIIP(tri) = EIIP(X) + EIIP(Y) + EIIP(Z)$$

Then the reweighted trinucleotide frequencies:

$$PseEIIP(tri) = EIIP(tri) \times f(tri)$$

All 64 trinucleotide combinations were summed to generate 64-dimensional features. This encoding method fuses local sequence patterns with physical energy distribution.

TNC (Trinucleotide Composition)

TNC is an encoding method that counts the frequency of all nucleotide combinations of length 3 in a nucleic acid sequence.

The normalized frequencies of all trinucleotide combinations are calculated as follows:

$$f(XYZ) = \frac{N(XYZ)}{L-2}$$

4^3 = 64 dimensional features were generated. For example, when the sequence length $L = 50$, each trinucleotide feature value is the number of occurrences divided by 48, which reflects the local context information of length 3.

RCk-mer (Reverse Compliment k-mer)

RCk-mer is a special encoding associated with k-mer. RC stands for reverse compliment related feature. In this experiment, $k = 5$, that is, the feature vector is generated by special processing of k-mer with length of 5.

First, calculate the k-mer and its reverse complement, (for example, “ACGTA”→“TACGT”)

Second, calculate the symmetric frequency:

$$f(kmersym) = \frac{N(kmer) + N(RC(kmer))}{2(L-k+1)} \quad (k = 5)$$

By merging the symmetric k-mer, the 1024 dimension is reduced to 512 dimensions, and the influence of sequence directionality is eliminated.

SCPseTNC (Series Correlation Pseudo TNC)

SCPseTNC is a pseudo-dinucleotide composition code that emphasizes the correlation information of trinucleotide sequences. This encoding takes into account both composition and long-range correlation.

Trinucleotide frequencies $f(tri)$ were first calculated:

Second, calculate the autocorrelation function for lag $\tau(\tau = 1{,}2)$:

$$\theta_\tau = \frac{1}{L-\tau-2}\sum_{i=1}^{L-\tau-2} \Phi(tri_i)\Phi(tri_{i+\tau})$$

Where $\Phi(tri)$ is the value of trinucleotide materialized attribute.

Generate pseudo-components:

$$SCPseTNC = [f(tri_i), \omega\theta_1, \omega\theta_2] \ \ (\lambda = 2)$$

In this experiment, the lag factor $\lambda = 2$, and weight $\omega = 0.1$.

Finally, 64+2 = 66 dimensional features were generated.

PCPseTNC (Physicochemical Pseudo TNC)

PCPseTNC ia a physicochemical related pseudo of three nucleotides encoding, is a combination of three nucleotide composition of frequency and the physical and chemical properties of the correlation.

First, the material property values $P_{k(tri)}$, $(k = 1{,}2{,}3)$ of the three nucleotides are calculated.

Second, the autocovariance of lagged attribute τ was calculated:

$$\Theta_{k\tau} = \frac{1}{L-\tau-2}\sum_{i=1}^{L-\tau-2} (P(tri_i) - \mu_k)(P(tri_{i+\tau}) - \mu_k)$$

Where μ_k is the mean of the attribute.

Generate pseudo components:

$$\mu_k = \frac{1}{L-2}\sum_{i=1}^{L-2} P_k(tri_i)$$

The number of physical properties d and chemical properties λ in this experiment (such as charge, hydrophobicity, stereo parameters) is 3 and 2 respectively.

Finally, 64 + 3×2 = 70 dimensional features were generated.

ANF (Accumulated Nucleotide Frequency)

ANF is an encoding of accumulated nucleotide frequency that calculates the cumulative nucleotide frequency at position j in the sequence:

$$ANF(j) = \frac{1}{j+1}\sum_{i=0}^{j}\delta(s_i, s_j) \quad (0 \le j \le L)$$

Where δ is the indicator function, which is 1 when s_i = s_j and 0 otherwise. L-Dimensional position-specific features are generated, reflecting the cumulative proportion of the current nucleotide s_j in the previous $j+1$ position of the sequence.

NAC (Nucleotide Composition)

NAC is a novel nucleotide composition encoding method, which considers the association information between nucleotides and not only focuses on the composition of individual nucleotides, but also pays attention to the relationship between them.

Global nucleotide composition frequencies were calculated as follows:

$$f(\alpha) = \frac{N(\alpha)}{L} \quad (\alpha \in \{A, C, G, T\})$$

The 4-dimensional feature was generated to describe the overall base preference of the sequence. For example, in a 100bp sequence with 30 ‘A’, $f(A) = 0.3$.

TAC (Tri-nucleotide Auto Covariance)

TAC encoding is used to calculate the autocorrelation information of trinucleotide in the sequence, which reflects the correlation of trinucleotide at different positions in the sequence. The auto-covariance of trinucleotide physicochemical properties was calculated as follows:

$$AC(\tau) = \frac{1}{L-\tau-2}\sum_{i=1}^{L-\tau-2}(\Phi(tri_i) - \mu)(\Phi(tri_{i+\tau}) - \mu)$$

A 4-dimensional feature was generated to describe the overall base preference of the sequence. For example, in a 100bp sequence with 30 ‘A’, $f(A) = 0.3$.

Where μ is the mean value of the attribute, which is expressed as follows.

$$\mu = \frac{1}{L-2}\sum_{i=1}^{L-2}\Phi(tri_i)$$

In this experiment, the lag factor $lag = 2$. The 16 physical and chemical attributes were calculated separately, and 16×2 = 32 dimensional features were generated to reflect the long-range correlation of trinucleotide attributes.

Cross-group k-mer spectrum analysis

The cross-group k-mer spectrum analysis on 4mC datasets includes 4 key steps:

(1) Calculated absolute k-mer frequencies (k = 3~5) in positive sequences per species using entire negative sequences as background;

(2) Aggregated k-mer counts within prokaryotic/eukaryotic groups to build joint frequency matrices;

(3) Identified significantly differential k-mers between groups via Fisher's exact test with directional frequency differences (positive - negative);

(4) Visualized top 10 FDR-corrected significant k-mers per k-value through heatmaps to reveal their group-specific distribution patterns.

EnDeep4mC Web Server Implementation and Usage

Software Architecture Overview

The EnDeep4mC web server implements a three-tier ensemble deep learning framework for DNA 4-methylcytosine (4mC) site prediction. The system employs Flask-based RESTful API architecture with TensorFlow 2.5 for deep learning model inference and scikit-learn for ensemble meta-learning.

Ensemble Model Structure

The prediction framework consists of three hierarchical layers:

(1) Base Model Layer: Parallel execution of CNN, Bidirectional LSTM, and Transformer architectures

(2) Meta-learning Layer: Integration of XGBoost and LightGBM classifiers

(3) Decision Fusion Layer: ElasticNet-based probability calibration

Feature Engineering Pipeline

The system incorporates 14 distinct encoding methods with dual-adaptive selection:

(1) Species-adaptive: Feature ranking optimized for each target organism

(2) Model-adaptive: Feature subsets tailored to individual base model requirements

(3) Supported encodings include ENAC, Binary, NCP, EIIP, k-mer (k=4), CKSNAP, PseEIIP, TNC, RCk-mer, SCPseTNC, PCPseTNC, ANF, NAC, and TAC

System Configuration and Parameters

(1) Server Specifications

Hardware requirements

CPU: 4+ cores recommended

RAM: 8GB minimum, 16GB recommended

Storage: 2GB for models and dependencies

Software dependencies

Python 3.7+

TensorFlow 2.4+

Flask 2.0+

scikit-learn 1.0+

Joblib 1.1+

(2) Runtime Configuration

```
# Server settings
app.config['MAX_CONTENT_LENGTH'] = 16 * 1024 * 1024    # 16MB file size limit
model_executor = ThreadPoolExecutor(max_workers=4)       # Model inference threads
feature_executor = ProcessPoolExecutor(max_workers=4) # Feature generation processes
# Supported species and models
SPECIES_LIST = ['4mC_A.thaliana', '4mC_C.elegans', '4mC_D.melanogaster',
                '4mC_E.coli', '4mC_G.subterraneus', '4mC_G.pickeringii']
BASE_MODELS = ['CNN', 'BLSTM', 'Transformer']
```

(3) Model Architecture Parameters

Deep Learning Components:

1)CNN: Three Conv1D layers with [64, 128, 256] filters, kernel sizes [7, 5, 3], ReLU activation, and MaxPooling1D

2)BLSTM: Bidirectional LSTM with [128, 64] units, 0.3 dropout, and 0.2 recurrent dropout

3)Transformer: 8 attention heads, 256 feed-forward dimensions, and 4 encoder layers

Ensemble Parameters:

1)XGBoost: max_depth=6, n_estimators=100

2)LightGBM: num_leaves=31, learning_rate=0.05

3)ElasticNet: alpha=0.1, l1_ratio=0.5

Input Specifications and Processing Workflow

(1) Sequence Requirements

Format: Standard FASTA format with optional headers

Sequence Length: 20 - 100,000 nucleotides

Valid Characters: A, T, C, G (case-insensitive)

File Encoding: UTF-8 text format

(2) Prediction Pipeline

1)Input Validation: Character validation, length checking, and duplicate removal

2)Feature Generation: Parallel execution of 14 encoding methods with species-specific selection

3)Base Model Inference: Individual predictions from CNN, BLSTM, and Transformer models

4)Meta-learning: Feature aggregation and ensemble prediction using XGBoost and LightGBM

5)Decision Fusion: Final probability calibration through ElasticNet regression

(3) Output Interpretation

Probability Scores: Continuous values from 0.0 to 1.0 representing prediction confidence

Classification Threshold: ≥0.5 classified as 4mC site, <0.5 as negative site

Result Formats: Interactive web visualization and downloadable text reports

Performance Characteristics

(1) Computational Efficiency

Single sequence prediction: ~2 seconds

Complete benchmark datasets: <3 minutes processing time

Parallel processing enabled for batch predictions

Feature caching mechanism for repeated sequences

(2) Accuracy Metrics

Accuracy (ACC): 0.9528 (range: 0.9133 – 0.9973)

Sensitivity (SN): 0.9557

Specificity (SP): 0.9499

Matthews Correlation Coefficient (MCC): 0.9056

Area Under the Curve (AUC): 0.9863 (range: 0.9697 – 0.9999)

F1-Score: 0.9529

Implementation Details

File Organization

```
EnDeep4mC/
├── web_server/app_5cv.py          # Main Flask application
├── pretrained_models/5cv/         # Model storage
│   ├── ensemble_5cv_*.pkl         # Ensemble models
│   ├── *_best_*.h5                # Deep learning models
│   ├── scalers/                   # Feature standardization
│   └── feature_configs/           # Feature selection
├── feature_engineering/           # Feature processing
└── prepare/                       # Data preprocessing
```

Deployment Instructions

Install Python dependencies from requirements.txt

Place pretrained models in designated directories

Execute python app_5cv.py from web_server directory

Access application at http://localhost:5000
