# Supplementary material for: EnDeep4mC predicts DNA N4-methylcytosine sites using a dual-adaptive feature encoding framework in deep ensembles
Source: Genome Res. 2026 Mar;36(3):589–99. doi: 10.1101/gr.280977.125 (PMC12951953; doi:10.1101/gr.280977.125)
Supplement: Supplement 1 [file Supplemental_Code.zip › Supplemental_Code/EnDeep4mC-main/web_server/templates/index.html]

EnDeep4mC WebServer


*EnDeep4mC*: A dual-adaptive feature encoding framework in deep ensembles for predicting DNA N4-methylcytosine sites

A deep learning ensemble predictor for DNA 4mC modification sites.
  
Combines CNN, BLSTM and Transformer models with advanced feature engineering.

- Home
- Predictor
- Model Architecture Details
- Example FASTA Samples
- ⬇️ Download Test Samples

Predictor

Target Species:

Arabidopsis thaliana
Caenorhabditis elegans
Drosophila melanogaster
Escherichia coli
Geobacter subterraneus
Geobacter pickeringii

DNA Sequence Input:

Or Upload FASTA File:

Analyze Sequences

Clear Form

### ℹ️ Variable-Length Sequence Processing

**EnDeep4mC** can process DNA sequences of any length (20-100,000 bp) using the following pipeline:

1. Length Standardization

All sequences are standardized to 41bp windows through appropriate padding or segmentation

2. Cytosine Filtering

Only windows with cytosine (C) at the center position are analyzed for 4mC prediction

3. Sliding Window Analysis

Long sequences are analyzed using overlapping 41bp windows with 1bp step size

4. Ensemble Prediction

Each 41bp window is analyzed by CNN, BLSTM, and Transformer ensemble

**Note:** For sequences longer than 41bp, predictions are averaged per cytosine position to provide robust methylation likelihood scores.

## Model Architecture Details

### Table 1. Architecture and Hyperparameters of Base Deep Learning Models

| Component | CNN | Bi-LSTM | Transformer |
| --- | --- | --- | --- |
| Input | (1, feature\_dim) | (1, feature\_dim) | (None, feature\_dim) |
| Layer 1 | Conv1D(256,1)+BN | BiLSTM(128) | MultiHead(8 heads) |
| Layer 2 | SepConv1D(128,3)+Pool | BN | FFN(512)+LayerNorm |
| Layer 3 | Conv1D(64,1) | BiLSTM(64) | 2 encoder layers |
| Pooling | GlobalMaxPool | - | GlobalAvgPool |
| Dense | Dense(128) | Dense(64) | Dense(128) |
| Output | Dense(1, sigmoid) | Dense(1, sigmoid) | Dense(1, sigmoid) |
| Regularization | L2(0.001), Drop(0.3) | L2(0.001), Drop(0.2), RecDrop(0.1) | L2(0.001), Drop(0.1) |
| Optimizer | Adam(lr=0.001, clip=1.0) | Adam(lr=0.001, clip=1.0) | Adam(lr=0.001) |

### Table 2. Configuration of Ensemble Learning Framework

| Component | XGBoost Configuration | LightGBM Configuration | Meta-Learner Configuration |
| --- | --- | --- | --- |
| Model Type | XGBClassifier | LGBMClassifier | LogisticRegression |
| Number of Trees | n\_estimators=500 | n\_estimators=300 | - |
| Learning Rate | 0.05 | 0.05 | - |
| Depth | max\_depth=7 | num\_leaves=63 | - |
| Regularization | gamma=0.1, subsample=0.8 | reg\_alpha=0.2, reg\_lambda=0.2 | C=0.6, l1\_ratio=0.5 |
| Others | colsample\_bytree=0.8 | min\_child\_samples=20 | penalty='elasticnet', solver='saga' |

### Model Architecture Diagram

Integrated Deep Learning Architecture with Dual-Adaptive Encoding System

### Example FASTA Format

```
>Sample_Sequence_1 (41bp)
ATGCTAGCTAGCTAGCTAGCTAGCTAGCTAGCTAGCTAGCTAGC
>Sample_Sequence_2 (50bp)
CGATCGATCGATCGATCGATCGATCGATCGATCGATCGATCGATCGATCGA
>Sample_Sequence_3 (30bp)
ATCGATCGATCGATCGATCGATCGATCGAT
```

**Variable-Length Examples:** The webserver accepts sequences of any length (20-100,000 bp).
Short sequences will be standardized to 41bp, and long sequences will be analyzed using sliding windows.

⬇️ Download Test Samples

*EnDeep4mC*: Ensemble Deep Learning for 4mC Site Prediction

Bioinformatics Research Group, UESTC  
If you have any questions, please contact:
frank.shuyu@outlook.com
